# The gut microbiota and diabetes: research, translation, and clinical applications – 2023 *Diabetes*, *Diabetes Care*, and *Diabetologia* Expert Forum

**DOI:** 10.1007/s00125-024-06198-1

**Published:** 2024-06-24

**Authors:** Mariana Byndloss, Suzanne Devkota, Frank Duca, Jan Hendrik Niess, Max Nieuwdorp, Marju Orho-Melander, Yolanda Sanz, Valentina Tremaroli, Liping Zhao

**Affiliations:** 1 Vanderbilt University Medical Center, Nashville, TN USA; 2 Howard Hughes Medical Institute, Vanderbilt University Medical Center, Nashville, TN USA; 3 Cedars-Sinai Medical Center, Human Microbiome Research Institute, Los Angeles, CA USA; 4 University of Arizona, Tucson, AZ USA; 5 Department of Biomedicine, University of Basel, Basel, Switzerland; 6 Department of Gastroenterology and Hepatology, University Digestive Healthcare Center, Clarunis, Basel, Switzerland; 7 Department of Internal and Vascular Medicine, Amsterdam University Medical Centers, Amsterdam, the Netherlands; 8 Amsterdam Diabeter Center, Amsterdam University Medical Centers, Amsterdam, the Netherlands; 9 Department of Clinical Sciences in Malmö, Lund University Diabetes Centre, Lund University, Malmö, Sweden; 10 Institute of Agrochemistry and Food Technology, Spanish National Research Council (IATA-CSIC), Valencia, Spain; 11 Wallenberg Laboratory, Department of Molecular and Clinical Medicine, Institute of Medicine, University of Gothenburg, Gothenburg, Sweden; 12 Department of Biochemistry and Microbiology, School of Environmental and Biological Sciences, Rutgers University, New Brunswick, NJ USA

**Keywords:** Butyrate, Faecal microbiota transplantation, Gastrointestinal microbiota, Gastrointestinal tract microbiology, Gut microbiota, Large intestine microbiota, Metagenomics, Microbiota metabolites, Review, Short-chain fatty acids, Small intestine microbiota

## Abstract

This article summarises the state of the science on the role of the gut microbiota (GM) in diabetes from a recent international expert forum organised by *Diabetes*, *Diabetes Care*, and *Diabetologia*, which was held at the European Association for the Study of Diabetes 2023 Annual Meeting in Hamburg, Germany. Forum participants included clinicians and basic scientists who are leading investigators in the field of the intestinal microbiome and metabolism. Their conclusions were as follows: (1) the GM may be involved in the pathophysiology of type 2 diabetes, as microbially produced metabolites associate both positively and negatively with the disease, and mechanistic links of GM functions (e.g. genes for butyrate production) with glucose metabolism have recently emerged through the use of Mendelian randomisation in humans; (2) the highly individualised nature of the GM poses a major research obstacle, and large cohorts and a deep-sequencing metagenomic approach are required for robust assessments of associations and causation; (3) because single time point sampling misses intraindividual GM dynamics, future studies with repeated measures within individuals are needed; and (4) much future research will be required to determine the applicability of this expanding knowledge to diabetes diagnosis and treatment, and novel technologies and improved computational tools will be important to achieve this goal.

**Supplementary Information:**

The online version contains a slideset of the figures for download available at 10.1007/s00125-024-06198-1.

## Introduction

In October 2023, a closed-door, day-long forum organised under the auspices of the journals *Diabetes*, *Diabetes Care*, and *Diabetologia* took place during the European Association for the Study of Diabetes 2023 Annual Meeting in Hamburg, Germany. The express goal of the forum was to create consensus and perform gap analyses to advance research into the role of the gut microbiota (GM) in diabetes. Discussions fell under four main headings: epidemiology; physiology and pathophysiology; technology and methodology; and clinical applications.

The group acknowledged that many of the gaps in understanding of the GM’s role in metabolic diseases are not unique to the diabetes field, but rather reflect broader needs to [1] conduct more well-controlled prospective and retrospective human studies that are followed up mechanistically with model systems studies and [2] refine computational tools and welcome a return of microbiology and molecular biology to our experimental toolkit. Nonetheless, there was agreement that the current reproduced microbiome data represent compelling target areas for future diabetes research. This article presents a distillation of the evidence and recommendations on important microbiome focus areas that would benefit from the attention of young and established diabetes researchers alike. Key knowledge gaps and challenges discussed in this article are summarised in the textbox (see ‘The GM and diabetes: key knowledge gaps and challenges’).


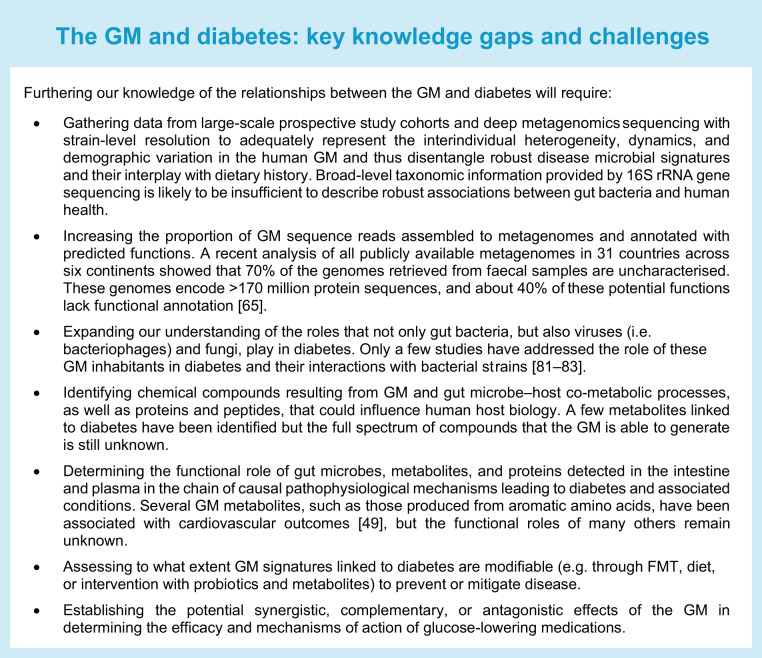


## Epidemiological perspectives

### Epidemiological associations between the GM and diabetes

**Fig. 1:**
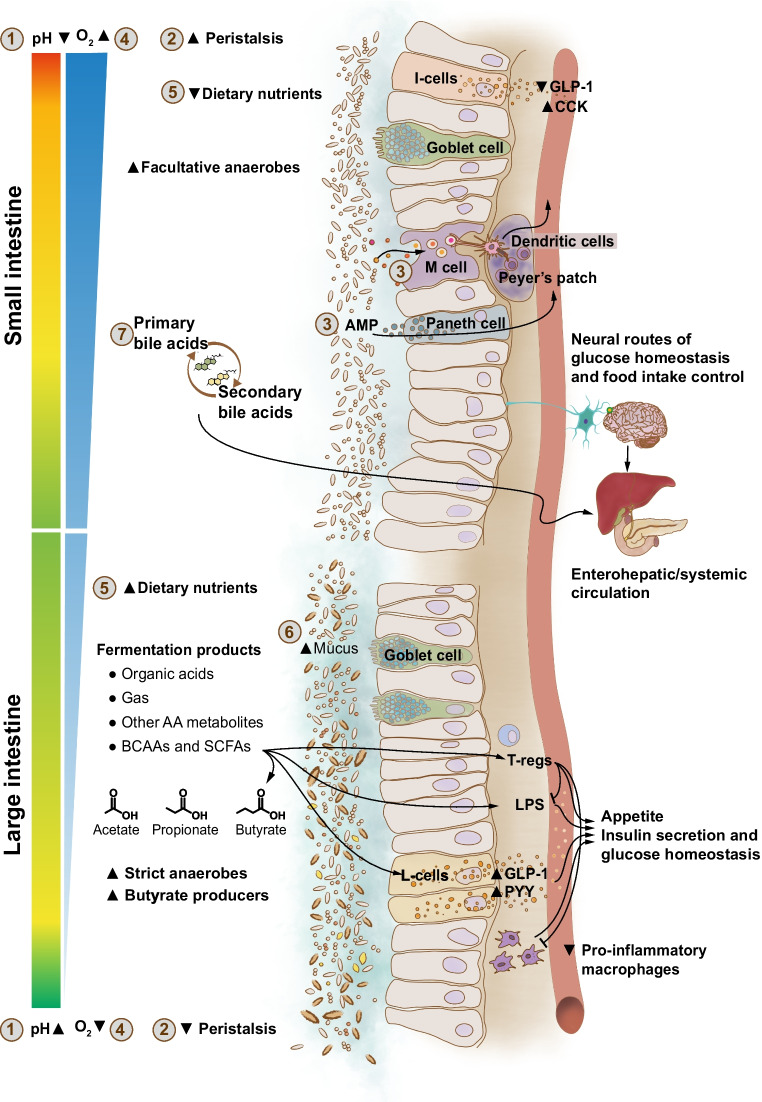
Microecological and physiological differences along the GI tract [103, 234]. Environmental conditions vary along the GI tract depending on physical, nutritional, and biological host factors, which translate into adaptations and differences in the intestinal bacteria inhabiting the different regions and their physiological functions through multidirectional interactions that may affect glucose metabolism and diabetes risk. The main factors affecting the microbial load and composition in the different regions are as follows. (1) pH values increase drastically from the stomach (pH 1.0–4.4) to the small intestine (pH 5.5–7.0) and then more progressively to the colon, where the pH can drop again (pH 5.5) as a consequence of the microbial fermentation of complex carbohydrates (fibre). The pH increases again in faeces (up to pH 7.8). (2) Intestinal transit is shorter and peristaltic movements are more intense in the small intestine than in the large intestine. (3) Small intestinal host epithelial cells (Paneth cells) secrete AMPs, acting as an innate defensive barrier reducing bacterial colonisation, and M cells of Peyer’s patches also pick up bacteria from the intestinal lumen. (4) Oxygen levels are also progressively reduced from the small intestine to the large intestine. (5) Dietary nutrients (proteins, lipids, and simple carbohydrates) are primarily digested by host enzymes and rapidly absorbed in the small intestine, limiting the accessibility of nutrients to intestinal bacteria; in contrast, partially undigested dietary residues (complex carbohydrates and partially hydrolysed proteins/amino acids) accumulate in the large intestine, where they serve as nutrients for bacteria. (6) Host glycans forming part of the mucous layer (produced by goblet cells), which is remarkably thicker in the large intestine than in the small intestine, also represent a nutrient source for intestinal bacteria, supporting their growth. (7) Bile acids are secreted to the small intestine, inhibiting and favouring the growth of specific bacteria that participate in their metabolism and recirculation. Altogether, those abiotic and biotic factors affect the ecological conditions that facilitate the survival of denser populations of bacteria moving to the most distal parts of the intestine (from 10^2^–10^4^ bacterial cells/g in the duodenum to 10^7^–10^9^ in the ileum and 10^11^–10^12^ in the colon) and account for differences in bacterial composition, with facultative anaerobes preferentially colonising the small intestine and strict anaerobes dominating the microbiota of the large intestine, including butyrate producers. In the large intestine, EECs, mainly L-cells, are stimulated by SCFAs (butyrate and propionate) to induce the hormones GLP-1 and PYY, which contribute to insulin secretion and glucose homeostasis and regulate appetite. In the small intestine, other EECs, such as I-cells, predominate and produce the hormone CCK, which induces digestive enzymes and bile and suppresses appetite. This is also the main region where nutrient signals are sensed by the enteric neurons and vagal afferents and thus signal to the brain to control energy homeostasis, although knowledge of the role of the gut microbiota in their regulation is limited. SCFAs, especially butyrate, can also induce immunoregulatory T cells (T-regs) that protect against obesity-induced pro-inflammatory macrophages and prevent LPS translocation. AA, amino acid; AMP, antimicrobial peptide; CCK, cholecystokinin; PYY, peptide YY. This figure is available as part of a downloadable slideset

The GM is the largest and most complex microbial community of the human body, connecting our external and internal milieu (Fig. 1). The motivation for epidemiological studies of the GM in obesity and cardiometabolic diseases, including type 2 diabetes, emerged from rodent studies demonstrating links among the GM, adiposity, and glucose tolerance [1, 2]. In humans, epidemiological studies have observed decreased microbial diversity in obesity, but no generalisable obesity-associated gut microbial signature has emerged from meta-analyses of small cohorts profiled by 16S rRNA gene sequencing [3, 4] or whole-genome metagenomics [5]. However, a large GM study using deep-sequencing whole-genome metagenomics in 34,057 individuals from Israel and the United States demonstrated consistent GM–phenotype associations and the predictive accuracy of machine-learning models trained on microbiome data for body mass index (BMI) and HbA_1c_ that could be replicated across the cohorts [6]. By subsampling the training cohort, these authors showed increased predictive accuracy with increased cohort size, with ~7500–10,000 individuals optimal for replicable results. This finding highlights the necessity of using large cohorts with hundreds of individuals and deep-sequencing whole-genome metagenomics that adequately represent the embedded interindividual heterogeneity and regional and demographic variation in human GM cross-sectional studies.

**Fig. 2:**
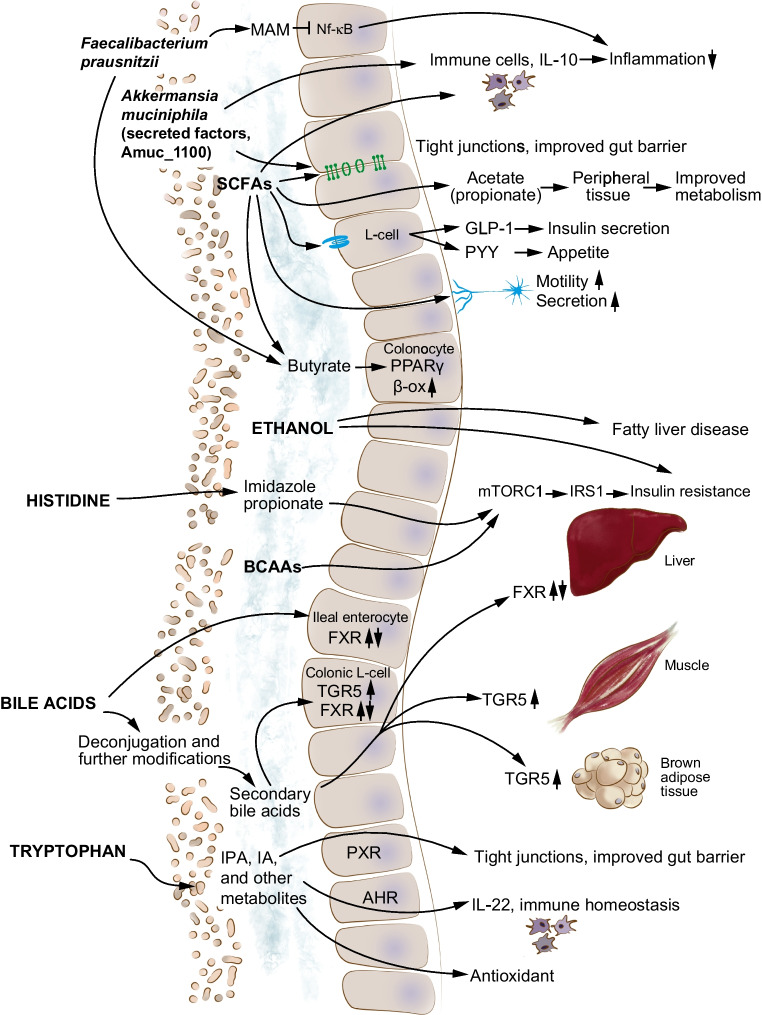
GM metabolites and signalling molecules linked to glucose metabolism and type 2 diabetes. Structural and secreted GM proteins are involved in the modulation of immune responses and inflammation, as shown for a protein secreted by *F. prausnitzii* (microbial anti-inflammatory molecule [MAM]), which is able to inhibit the nuclear factor-κB (Nf-κB) pathway. Another example is Amuc_1100, an outer membrane protein of *A. muciniphila*, which improves the gut barrier and decreases inflammation. The GM also produces SCFAs, which stimulate the release of incretin hormones and improve peripheral tissue metabolism. In addition, SCFAs modulate immune cell function, improve the gut barrier, and stimulate enteric neuron signalling. The SCFA butyrate also provides energy to colonocytes and increases colonocyte β-oxidation (β-ox) by activating peroxisome proliferator-activated receptor-γ (PPARγ). Bile acid signalling through the bile acid receptors FXR and TGR5 modulates metabolic responses in several different tissues. GM tryptophan metabolites, such as indolepropionic acid (IPA) and indoleacrylic acid (IA), modulate immune and metabolic responses by improving the gut barrier through the pregnane X receptor (PXR) and by signalling through the aryl hydrocarbon receptor (AHR) on intestinal immune cells and increasing the production of interleukin-22 (IL-22). In the bloodstream, IPA and IA also provide antioxidant and anti-inflammatory functions. Imidazole propionate and BCAAs impair insulin signalling through activation of the mechanistic target of rapamycin complex 1 (mTORC1). The GM also produces ethanol, which is linked to fatty liver disease and insulin resistance. IL-10, interleukin-10; IRS1, insulin receptor substrate 1; PYY, peptide YY. © 2019 Canadian Diabetes Association. Adapted from Caesar [235] with permission from Elsevier. This figure is available as part of a downloadable slideset

Several observational studies have reported associations between the GM and type 2 diabetes. Consistent features of altered GM composition in type 2 diabetes and impaired glucose tolerance/fasting glucose, found in epidemiological studies worldwide and also occurring in the metabolic syndrome, are reduced diversity and decreased abundance of bacteria that produce the short-chain fatty acid (SCFA) butyrate (Figs 1 and 2) [7–14]. Some studies have also observed an increase of opportunistic pathogens [7, 8, 11], some of which have been linked to subclinical coronary atherosclerosis [15]. The mucus-degrading bacterium *Ruminococcus gnavus*, which has been linked to inflammatory bowel diseases, recently has also been identified as a predictor of several features of the metabolic syndrome, including low-grade inflammation, large waist circumference, elevated serum triglycerides, elevated HbA_1c_, and decreased HDL-cholesterol [16]. However, as indicated by meta-analyses of GM alterations across different diseases, including gastrointestinal (GI) and metabolic diseases, several of these features are not disease-specific and might characterise a general GM dysbiosis [4, 5]. Therefore, to disentangle disease-specific microbial signatures beyond differences in race/ethnicity, lifestyle, and other demographic characteristics, it will be important to perform studies in large populations and to include healthy individuals/control participants from different studies as references; these approaches have been shown to increase disease prediction accuracy [5, 6].

In addition to a decreased capacity to produce butyrate, GM functional capabilities that are altered in type 2 diabetes are involved in the production of branched-chain amino acids (BCAAs) and the metabolism of B vitamins and simple sugars [7, 12, 17, 18]. Increased levels of circulating BCAAs have been described in insulin-resistant individuals and linked to a higher risk of type 2 diabetes [19]. In line with this, an increased potential to synthesise BCAAs and decreased microbial BCAA uptake and catabolism have been described in the GM of insulin-resistant individuals with normoglycaemia [18]. However, the analyses of GM functions only show an altered potential. Quantification of metabolites has recently been performed to validate these findings. Figure 2 depicts the links between GM metabolites and signalling molecules that have been observed for glucose metabolism and type 2 diabetes.

#### Associations between the GM or its metabolites and glucose-lowering drug treatments

Evidence supporting the role of the GM in type 2 diabetes has been strengthened by observational and interventional studies demonstrating changes in the relative abundance of multiple bacterial species in the GM of metformin users [10, 20–22]. A higher relative abundance of *Escherichia coli* and a decreased abundance of *Intestinibacter bartlettii* [10, 20, 21] have been described in multiple cohorts involving individuals being treated with metformin. Additionally, an increase in *Escherichia marmotae* and a decrease in *Romboutsia timonensis* have been found in metformin-treated individuals in a recent large metagenomic study [23].

**Fig. 3:**
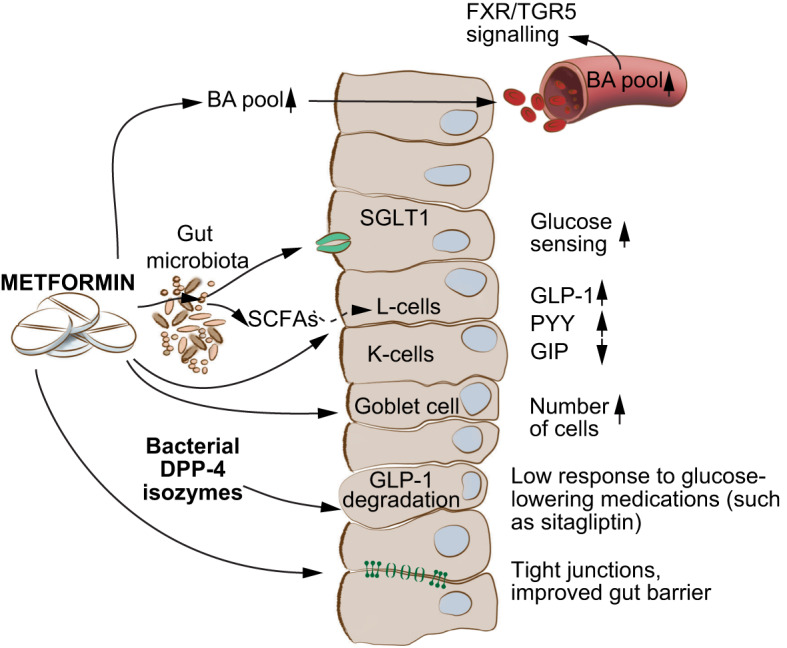
GM interactions with glucose-lowering medications. GM metabolites are involved in the mechanism of action of metformin, including bile acid (BA) signalling through the bile acid receptors FXR and TGR5 and production of SCFAs, which modulate the release of incretin hormones such GLP-1, gastric inhibitory polypeptide (GIP), and peptide YY (PYY) from enteroendocrine cell (K- and L-cells). Other GM-dependent mechanisms involved in the action of metformin include improved glucose sensing through sodium–glucose cotransporter 1 (SGLT1) and an improved gut barrier (e.g. restoration of tight junctions and increase in mucin-producing goblet cells [236, 237]). However, through the expression of DPP-4 isozymes, the GM might decrease GLP-1 activity and affect the efficacy of glucose-lowering drugs. © 2019 Canadian Diabetes Association. Adapted from Caesar [235] with permission from Elsevier. This figure is available as part of a downloadable slideset

Support for the causal effects of these GM differences in type 2 diabetes has been provided by randomised trials and studies in drug-naive individuals demonstrating that the GM compositional changes translate to enhanced production of propionate and butyrate [20, 21] and modulation of bile acid pools [21], which may mediate some of the glucose-lowering effects of metformin (Fig. 3) [20–22, 24]. However, the GM might also be responsible for the transient or persistent intestinal discomfort experienced by ~30% of individuals who take metformin (e.g. through increased gas production by some *Escherichia* species) [22, 25].

With regard to other oral glucose-lowering drugs, studies have shown effects of dipeptidyl peptidase 4 (DPP-4) inhibitors and α-glucosidase inhibitors on the GM and microbial metabolites, but less clear effects of sodium–glucose cotransporter 2 (SGLT2) inhibitors, thiazolidinediones, and glucagon-like peptide 1 (GLP-1) receptor agonists [26–28].The majority of studies to date involving SGLT2 inhibitors have been conducted in mouse models, and the few existing human studies have provided contradictory results and were unable to clearly discriminate the effects of the SGLT2 inhibitor from those of previous or concomitant metformin treatment or concurrent lifestyle modifications [29]. GLP-1 receptor agonists may exert anti-inflammatory effects (e.g. through activation of the intraepithelial lymphocyte GLP-1 receptors), which in turn could contribute to modulating the gut microbiota [30, 31]. Although much more research is needed, existing evidence suggests that the GM may mediate some of the benefits of glucose-lowering treatments [26], and certain probiotics or prebiotics might further improve the glucose-lowering effects of these drugs through their effects on the GM or its functions [32]. Further interventional and translational studies are needed to determine whether drug-induced GM changes are causally involved in mediating health effects and to uncover the underlying mechanisms.

Importantly, the GM might also influence the efficacy of glucose-lowering drugs, for example by expressing homologues of human DPP-4, which can decrease the activity of GLP-1 and affect glucose metabolism (Fig. 3) [33, 34]. Because bacterial DPP-4 homologues seem resistant to some drugs targeting human DPP-4 [33], inhibition of bacterial isozymes might be required to improve metabolic responses to current medications.

#### Associations between GM metabolites and diabetes-related traits

##### SCFAs

The GM ferments plant-based dietary carbohydrates and fibre, as well as peptides that reach the large intestine, to produce SCFAs—mainly acetate, propionate, and butyrate. After hepatic metabolism, ~70% of colonic acetate, but only small amounts of propionate and butyrate (<2% for butyrate), reach the circulation [35]. As described in more detail in ‘Physiological and pathophysiological perspectives’, SCFAs regulate several processes, including intestinal motility and pH, gut barrier immune responses and systemic metabolism through pathways affecting gluconeogenesis, insulin sensitivity, and insulin secretion (Fig. 2) [36]. However, human studies show extensive variation in the levels of different SCFAs in the stools and/or blood of individuals with type 2 diabetes, which is likely because of methodological limitations [36]. The strongest support for the role of SCFAs in the regulation of glucose metabolism is provided by animal studies and one recent human study using the Mendelian randomisation (MR) statistical method [36, 37] (see ‘Role of MR in elucidating causal effects’ below).

##### Bile acids

Bile acids are amphipathic molecules that mediate the absorption of dietary fats and lipid-soluble vitamins. These molecules are also recognised as major players in regulating lipid, glucose, and energy metabolism. Consequently, alterations in bile acid pools have been found in type 2 diabetes and other obesity-related diseases and identified as possible contributors to the pathophysiology of type 2 diabetes (Fig. 2) [24, 38–41].

Increased levels of 12α-hydroxylated bile acids [41] and decreased levels of 6α-hydroxylated bile acids [42, 43] are linked to insulin resistance and occur in people with type 2 diabetes. Increased levels of 6α-hydroxylated bile acids are observed after gastric bypass surgery and can predict type 2 diabetes remission [42]. The GM can deconjugate and transform bile acids, thus contributing to a highly variable but important portion of human bile acid pools (Fig. 1) [44]. For example, circulating levels of 6α-hydroxylated bile acids are found to co-vary with levels of specific *Clostridia* species in the gut [43].

Intervention studies have also investigated the potential importance of bile acids in human metabolism. Elevated systemic bile acid levels and intestinal signalling to stimulate the release of GLP-1 have been demonstrated after bariatric surgery, with postprandial increases found to be particularly important [45]. However, exaggerated bile acid responses have been found in some individuals with cholecystectomy and are associated with further increased GLP-1 and insulin responses [46]. In people with type 2 diabetes, metformin has been shown to improve glucose metabolism via a decreased abundance of *Bacteroides fragilis*, which has been linked to increased levels of glycoursodeoxycholic acid in the gut and inhibition of the farnesoid X receptor (FXR) [24]. However, our understanding of direct interactions between the GM and bile acids and their associations with the development and treatment of type 2 diabetes and related diseases is still limited, and more human studies are warranted.

##### Other metabolites

GM-produced amino acid metabolites have also been linked to type 2 diabetes (Figs 1 and 2). Increased circulating levels of 3-indolepropionic acid, a tryptophan catabolite, have been associated with improved insulin secretion and sensitivity and a decreased risk of type 2 diabetes [47]. Furthermore, increased plasma levels of imidazole propionate, a bacterial product of histidine metabolism, have been reported in individuals with insulin resistance and type 2 diabetes [48]. These metabolites and several others derived from GM catabolism of aromatic amino acids have also been associated with incident cardiovascular risk and mortality in independent cohorts from Europe and the United States [49, 50]. Finally, GM ethanol production has been associated with fatty liver disease [51–53] and might be linked to insulin resistance (Fig. 2).

#### Role of MR in elucidating causal effects

The GM can affect and interact with host health in numerous ways, and the arrow of causality is often bidirectional or even multidirectional. GM features at different levels (e.g. community, species, pathway, gene, and metabolite) can affect a host phenotype (e.g. altering the risk of obesity), while the development of a phenotype (e.g. obesity) can, in turn, change the GM.

MR is a statistical method that uses human genetic variants related to exposures to discriminate causal effects on disease outcomes from associations that result from confounding, reverse causation, or something else. To apply MR to investigate the connection between the GM and type 2 diabetes, the GM feature in question needs to be affected by a human genetic variant or multiple variants strongly enough to allow their use as instruments in instrumental variant analysis.

Although several genome-wide association studies have been performed on different GM features such as gut bacterial taxa relative abundances and human faecal microbial metabolites, large MR studies investigating their causal role in type 2 diabetes have been limited and are not yet confirmed in replication studies. Sanna et al [37] identified human genetic variants that associate with faecal SCFA levels and reported evidence for a potential causal connection between the GM’s butyrate production potential (i.e. genes responsible for GM butyrate production) and improved response to insulin during an oral glucose tolerance test. These authors also found a causal link between abnormal faecal propionate levels and increased type 2 diabetes risk [37]. Another MR study reported that type 2 diabetes and kidney disease increased plasma levels of the GM-dependent metabolite trimethylamine oxide (TMAO) and proposed that the earlier observational evidence of elevated risk of cardiovascular diseases with higher TMAO levels might have been the result of confounding or reverse causality rather than a causal effect [54]. Another recent study suggested that certain bacterial genera could have a causal link to type 2 diabetes [55]. Considering the limitations of both MR (e.g. pleiotropy and problems related to weak instrumental variables) and GM research (e.g. methodological differences, interindividual heterogeneity, and intraindividual variability), large, high-quality studies are needed to assess the ability of host genetic variants using MR to mimic specific GM features—whether specific bacterial species, genera, or metabolite products—to understand causal connections with type 2 diabetes pathogenesis.

#### Associations among diet, GM, and diabetes

Decreased dietary fibre intake has been associated repeatedly with increased risk of type 2 diabetes; accordingly, new dietary recommendations for diabetes management encourage high consumption of minimally processed plant foods such as whole grains, vegetables, whole fruit, legumes, nuts, and seeds [56]. Diet is a driver of the GM ecosystem, and microbially accessible carbohydrates promote GM diversity and SCFA generation, which decreases inflammation and supports the maintenance of the gut barrier [57].

In relation to the GM and glucose metabolism, increased fibre intake has been associated with increased levels of distinct species, for example *Prevotella copri* [58] (now renamed *Segatella copri*). Studies have also shown that the beneficial effects of fibres on HbA_1c_ may be mediated by the specific baseline GM composition and diversity of fibre-promoted SCFA-producing bacteria [59]. However, variable effects are observed even in well-controlled dietary interventions [60], and given the high interindividual variability of the GM, dietary responses of the GM are highly individualised [61]. Precision, or ‘personalised’, nutrition is an evolving field based on identifying individual-specific response-predictive features that can be used to design dietary interventions [62]. Using personal data on GM composition and other information such as blood biomarkers and dietary habits, machine-learning approaches have been applied to predict postprandial glycaemic responses to standardised meals with greater accuracy than other predictive methods [63, 64]. These studies have revealed that the specific composition of the GM contributes to the specific response of its host (i.e. the response to the diet differs in the presence of different bacteria). Hence, the GM determines, at least in part, metabolic heterogeneity among humans. Being modifiable and highly metabolically active, the GM offers possibilities for more precise lifestyle interventions and novel treatments.

### Knowledge gaps, challenges, and possibilities

Several large, high-quality reference genome catalogues now exist [65–67] and greatly facilitate taxonomic assignment and functional characterisation of the GM in human studies. However, these databases are not without limitations (see textbox). For epidemiological analyses, GM data are fraught with challenges, including great inter- and intraindividual variability, high dimensionality (i.e. the number of observed GM features may be larger than the number of samples and subjects), and sparsity (i.e. GM features such as species are only detected in part of the samples) [68]. At the population level, the GM is composed of thousands of interacting species, each harbouring genetic diversity across hosts and within a host over time; yet, commonly performed analyses often ignore such non-independence, the complex additive and interaction effects among the microbes, and the modifiability and fluctuations of the GM. However, some recent analyses have revealed different patterns of intraindividual variation and adaptation to host physiology for different bacterial species [14, 69, 70].

Other challenges relate to the remarkable number of phenotypic and environmental factors that the GM may influence and to which it may respond. The requirement of large cohorts has unquestionably been demonstrated in human genetics; most polygenic traits are known to be affected by many genetic variants with small effect sizes, which nonetheless can be summed to powerful polygenic risk scores of clinical importance [71, 72]. Similarly, as evidenced by the findings of the large metagenomic study from Israel and the United States [6], single bacterial species might have associations of low effect size with human phenotypes or be present in low abundance. Thus, large sample sizes for adequate statistical power and coverage of interindividual variability are necessary to obtain replicable results and high prediction accuracy.

To better understand the long-term influence of GM variation and dynamics on type 2 diabetes, prospective studies are crucial. In the few prospective studies published to date, GM features have been associated with incident type 2 diabetes in a geographically diverse Chinese population [73] and a subset of a Spanish clinical trial [74], both studies using 16S rRNA gene sequencing. GM features were also linked to type 2 diabetes in a large population-based Finnish cohort with 18 years of follow-up using shallow metagenomic sequencing [75]. However, in these studies, the number of incident cases was restricted, and the analyses had limited resolution (i.e. were restricted to the most dominant GM taxa), as none of the studies used deep-sequencing whole-genome metagenomics.

The importance of subspecies- and strain-level resolution in metagenomic studies may have been undervalued and is an important limitation to harnessing the GM for human health. For example, *Faecalibacterium prausnitzii* is among the most promising candidates for next-generation probiotics, but there are also other promising candidates, such as *Akkermansia muciniphila* and *P. copri* [76]. With regard to *F. prausnitzii*, several potential subspecies have been found in the human gut, harbouring different functional potential for the use of complex polysaccharides [77]. In line with this observation, several *F. prausnitzii* subspecies were also identified in the large metagenomic study from Israel and the United States, and negative associations with BMI were observed only for a subset of them [6]. In the case of *P. copri*, both positive and negative associations have been found with the host metabolic phenotype (e.g. visceral fat and glucose responses).

These inconsistent findings could be partially explained by intra- and interspecies diversity [78, 79]. The current code of nomenclature defines bacterial species based on genome similarity, with conspecific genomes having ≥70% similarity by DNA–DNA hybridisation and an average nucleotide identity ≥94% in the core genome and ≥96% in universal marker genes [80]. However, these genomic variations can translate into important phenotypic differences. For example, these differences may define a strain within the same species as commensal or pathogenic, as in the cases of *B. fragilis* and *Clostridium difficile*, depending on whether the strain encodes virulence factors [80]. Overall, the studies mentioned above demonstrate that differences at the strain or even substrain level are highly meaningful, and low-resolution analyses (such as 16S rRNA gene sequencing) miss key information.

Another knowledge gap concerns the viral component of the GM, predominantly comprising viruses that infect bacteria, known as bacteriophages (or, more simply, ‘phages’). Although these phages have not been well studied in the epidemiological setting, they may be important for understanding the bacterial dynamics of the GM that may affect its interactions with the host. To date, only a few epidemiological studies have reported associations between the gut phageome and type 2 diabetes [81, 82] or the metabolic syndrome [83]. Although initially promising, the conclusiveness of the results is limited because of the restricted sample sizes. Future studies of the role of phages as regulators of the GM and cardiometabolic health are warranted but will face challenges related to, among other issues, virome isolation and the limitations of current databases [84, 85].

Integrative multi-omics studies might be needed to investigate the intricate connections among environmental factors, the GM, the virome/phageome, and cardiometabolic phenotypes. Some pioneering examples of reasonably large studies have recently demonstrated the power of such approaches [14, 86–88]. The interactions are multifactorial and multidirectional and demand untargeted, large-sized, multi-omics and longitudinal approaches of high depth and resolution.

## Physiological and pathophysiological perspectives

### Current understanding of the role of the GM in the pathophysiology of diabetes

During their evolution, mammals had to adapt to a world rich in microbes, viruses, and fungi [89]. During and immediately after birth from a sterile intrauterine environment, mammals are exposed to potentially harmful microbes [90–92]. Evolution has created substantial barriers, including the GI transit process [93], immunoglobulin A (IgA) [94–96], mucus [97], the epithelial layer [98], the endothelial barrier [99], lymph nodes [100], and the liver [101], all of which prevent microbial translocation into the body but create an optimal reservoir for the microbial ecosystem [102]. Low microbe numbers are present in the upper GI tract. At the same time, high microbial density and richness are observed in the large intestine, along with physiological changes in the pH and aerobic/anaerobic conditions from the small to the large intestine, with anaerobic conditions in the large intestine (Fig. 1) [103].

#### Essential functions of microbes

Besides being a potential deleterious threat for mammals, gut microbes also provide essential functions for mammals, including the education of the immune system, protection from pathogens (i.e. colonisation resistance) [104], metabolic functions, and the supply of nutrients (e.g. vitamins [105]), gut motility, and detoxification of xenobiotics [106]. At the same time, there is competition between microbes and the host for nutrients in the small intestine, and microbially produced macronutrient byproducts are provided to the host. Nutrients (i.e. fibres) and mammalian metabolites such as glucuronides, mucous polysaccharides, and bile acids are fermented or transformed by microbial metabolism (Fig. 1) [107]. Microbial metabolism and microbial cell death and turnover contribute to pools of microbial metabolites in the peripheral blood, where ~30% of all peripheral blood metabolites show associations with the GM and its genes [108, 109]. These microbial metabolites are recognised by receptors such as G-protein-coupled receptors (GPCRs) [110] or the aryl hydrocarbon receptor (AHR) [111] or are further processed by mammalian enzymes such as TMAO to regulate mammalian gene expression by epigenetic modifications, with implications for cardiometabolic health [112–115].

#### Roles of non-digestible fibres and their metabolites

Non-digestible carbohydrates are an energy source for specific bacteria in the large intestine that contain enzymes, lacking in the host, that metabolise these fibres and promote the production of SCFAs. Numerous studies have demonstrated that exogenous administration of SCFAs, particularly propionate and butyrate, is beneficial in rodent models of diabetes-like phenotypes [116–118]. However, the evidence from clinical trials in both type 1 and type 2 diabetes is less clear [119–123].

In the colon, SCFAs activate enteroendocrine cells (EECs) via binding to GPCRs and free fatty acid receptors 2 and 3 to induce the release of gut peptides, mainly GLP-1 and peptide YY (Fig. 1) [124]. In support of this finding, supplementation with prebiotics in rodents and humans, which can improve glucose tolerance and insulin resistance, has been associated with increased levels of gut peptides [125]. In one study, a high-fibre diet improved glucose tolerance in individuals with type 2 diabetes, an effect that was associated with increased faecal butyrate levels and circulating GLP-1 [59]. GLP-1 regulates glucose homeostasis by increasing insulin secretion, promoting insulin sensitivity, and reducing hepatic glucose production (Fig. 1).

Additionally, SCFAs are crucial for maintaining overall gut health and the gut barrier, as butyrate is the primary fuel source for colonocytes. In contrast, reduced butyrate drives colonocytes toward anaerobic glycolysis, which increases epithelial oxygenation, disrupting the anaerobic environment of the colon [126]. Although SCFAs can act to increase gut peptide release or improve the gut barrier, additional work has highlighted a glucoregulatory role via their action in intestinal gluconeogenesis and on energy expenditure via brown adipose tissue, as well as direct action at the liver, pancreas, and even brain, all of which requires further exploration [127–131].

The GM produces a plethora of metabolites in addition to SCFAs, which likely play a crucial role in host glucose homeostasis (Fig. 2) [132]. For example, bile acids are known glucoregulatory signalling molecules, and their affinity for both FXR and Takeda GPCR 5 (TGR5) is significantly affected by deconjugation and metabolism into secondary bile acids coordinated by gut microbes [133–136]. Additionally, the GM converts tryptophan and other nutrients into indoles that act via the AHR to reduce inflammation in the metabolic syndrome, especially at the gut level [137, 138]. Furthermore, other gut-derived molecules such as TMAO and imidazole propionate have been implicated in the development of diabetes [139, 140].

#### Role of the GM in gut barrier functioning

The GM plays a vital role in gut barrier functioning. Impairment in the gut barrier leads to a leaky gut, contributing to low-grade systemic inflammation, a characteristic of obesity and diabetes [141, 142]. Although the mechanisms have been studied mostly in experimental models, one potential mechanism contributing to systemic inflammation is an increase in circulating lipopolysaccharide (LPS) endotoxins derived from the cell envelope of Gram-negative bacteria, also known as metabolic endotoxaemia (Fig. 1). LPSs can act on a specific pathogen-associated molecular pattern (PAMP)—toll-like receptor 4 (TLR4)—throughout the body to elicit a pro-inflammatory immune response that negatively affects glucose homeostasis. A series of studies have suggested a potential role for *A. muciniphila* in mediating some of the effects of alterations in the GM on systemic inflammation through actions on TLR4 and the gut barrier; however, less evidence is available on its role in mediating effects on glucose metabolism in metabolic disease [143–146]. However, much more research is needed to determine whether metabolite sensing by PAMPs other than TLR4 is implicated in regulating host–microbe crosstalk and gut barrier integrity [147] in humans.

In parallel, the accumulation of pro-inflammatory macrophages (Fig. 1), CD8αβ T cell infiltration, and reduced IgA+ immune cells are observed in the intestines of individuals with obesity [148–150], contributing to insulin resistance [149, 150]. GM modulation strategies could mitigate the adverse gut immune effects of hypercaloric diets. For example, reducing the proportion of pro-inflammatory macrophages and increasing type 3 innate lymphoid cells and regulatory T cells are associated with improved glucose metabolism. Nonetheless, understanding the precise molecular mechanisms driving microbe–immune interactions in the gut and their translation to humans will also require extensive future research.

### Knowledge gaps, challenges, and possibilities

Reductionist approaches are required to progress from correlations of bacterial phylotypes/strains with metabolic phenotype (e.g. diabetes) to mechanistic and causal relationships. However, many of the earlier analyses performed in epidemiological studies do not have sufficient resolution (i.e. 16S rRNA gene short amplicon sequencing only provides accurate identification at the genus level but not at the species, strain, and functional levels) [151], and deep-sequencing whole-genome metagenomics, coupled with strain-level analyses, are needed to identify bacteria and their functions that are linked to diseases and thereby to correctly downsize further causality and mechanistic studies [152].

The complexity of the GM and the multitude of possible phylotypes and microbial networks associated with specific phenotypes in population studies also make it difficult to test all possible hypotheses experimentally. Advanced statistics (e.g. MR and mediation analysis) and machine-learning methods are helping to establish causal inferences in human studies, but further validation stages are still needed to provide direct evidence of causality. For this purpose, the use of rodent models in which confounding variables (e.g. genetic background, microbial communities, and diet) can be controlled is key to clearly identifying the effects caused by the precise microbe investigated (namely, a bacterial strain or metabolite).

The use of whole faecal microbiota transplantation (FMT) or co-housing experiments in which the microbiota of rodents with different phenotypes are inter-exchanged have substantially helped to unravel cause and effect in whole communities of microorganisms [2, 153]. Nonetheless, the results of those trials could be biased by limitations in study design. For example, bias could arise in relation to the limited number of donors selected and the possible variations in rodent response to microbes from a different donor species (e.g. lack of colonisation or persistency of part of the microbial population or failure in replicating host–microbe interactions of the original host in which the microbiota co-evolved) and consequent differences in physiological effects [154].

Moreover, identifying the key microbial actors driving a health or disease phenotype and the non-active players within the community is a rather complex undertaking. Specific components of the GM could play a role per se or in coordination with other community members [155]. Furthermore, correlations and causal relationships between GM components and disease could also depend on the specific host (e.g. disease predisposition) and environmental context (e.g. dietary patterns), which can vary in different geographical locations and population groups [11].

One reductionist approach that could aid in establishing the causal role of microbes in metabolic disease is the use of defined microbial communities in gnotobiotic mouse models of diabetes [156]. This approach consists of assembling a defined community of well-characterised and genetically tractable microbes, which is then used to colonise gnotobiotic mice. This so-called ‘defined microbiota’ approach allows for the manipulation of a specific microbial feature in the background of a complex, yet manageable, microbial community to determine whether specific microbial functions play a causative role in the pathogenesis of diabetes and related complications.

Intervention studies with specific bacteria in the ecosystem, as well as more sophisticated strategies that deplete specific microbial components or functions, are ideal for providing evidence of causality [157, 158]. However, even if a particular bacterial genus and species has been correlated with a specific disease phenotype in a relatively reproducible manner in epidemiological studies [159] and proven to be causally involved in an intervention trial, differences between species, and even between strains, may also lead to different outcomes. As explained above, small genomic and phenotypic differences between strains belonging to the same species can translate into functional differences affecting the host phenotype (e.g. with regard to their immunomodulatory effects) [160]. Therefore, the results of efficacy and mechanistic analyses deduced from studies performed with a specific strain cannot be systematically generalised to all strains of the given species.

Historically, the study of the impact of GM on human disease has been focused on the large intestine microbiota because the human colon is the site in the body with the highest abundance of microbes and the most accessible intestinal section. The contribution of the large intestine microbiota to the pathogenesis of metabolic disease has been demonstrated by FMT studies in mouse models of obesity and related complications [2, 153]. However, it is important to note that the small intestine overshadows the large intestine with regard to metabolic regulation. The small intestine is home to EECs that produce GLP-1 and other incretin hormones, which are key glucose metabolism regulators [161]. The small intestine epithelium also plays an essential role in glucose and fat uptake and metabolism, protecting the host from features of metabolic dysfunction [162, 163], and the small intestine microbiota is a regulator of EEC function and nutrient absorption, metabolism, and secretion [164, 165]. Thus, microbiota–host interactions in the small intestine would be expected to contribute to diabetes pathogenesis. Recent work in mouse models has determined that specific members of the small intestine microbiota can inhibit lipid secretion by enterocytes and limit serum triglyceride concentrations during the consumption of a Western-style obesogenic diet [166]. Additionally, the small intestine microbiota in rodents has been demonstrated to affect nutrient-induced gut–brain signalling, which regulates glucose homeostasis [167, 168].

Despite their importance, host–microbiota interactions in the small intestine and their relevance to diabetes are understudied because of limitations in the process of acquiring small intestine microbiota samples from humans and the reduced abundance of microbes in this portion of the digestive tract. Novel technologies to overcome technical limitations in the study of the small intestinal microbiota are discussed further in ‘Technological and methodological advancements’.

GM fluctuation, especially related to dietary intake, should be considered when establishing what are normal and what are dysfunctional microbiota changes for metabolic health. The GM mirrors individuals’ habitual diet and daily choices. Therefore, longitudinally considering dietary history and GM variations over multiple days could help to fine-tune associations and infer causal relationships regarding the metabolic health of individuals [169]. Moreover, daily oscillations of the GM related to eating patterns also affect its functional roles, such as appetite regulation and postprandial responses to food intake, with potential long-term effects on metabolic health and diabetes risk. For example, mouse studies have shown that daily oscillations in GM composition are required to maintain the circadian release of GLP-1, which in turn is required to achieve appropriate circadian control of metabolic homeostasis [170]. In humans, type 2 diabetes and obesity are correlated with alterations of GM circadian rhythms [171], suggesting that daily oscillations are relevant to understanding the role of the GM in controlling energy homeostasis.

Sex also seems to contribute to GM variations, although its relevance for predicting health associations and their underlying mechanisms are under-investigated. The epidemiology and pathophysiology of obesity and associated cardiometabolic disorders such as type 2 diabetes have a sex dimorphism that may be related to not only the role of sex hormones in fat distribution, metabolism, and immunity, but also differences in the GM [172]. Studies in mouse models suggest reciprocal interactions between sex hormones and the GM. On one hand, the GM may regulate the production and/or metabolism of sex hormones (i.e. testosterone and oestrogens), as proven in a non-obese diabetic mouse model of type 1 diabetes [173]. On the other hand, physiological effects of sex hormones (e.g. on immunity and intestinal transit) may affect the GM [174]. Therefore, sex should be considered a confounding variable in epidemiological studies and in the design of mechanistic studies using mouse models because, to date, most preclinical studies have been carried out exclusively in males.

## Technological and methodological advancements

Separating phenomenology from actual biology in the microbiome field requires tools and approaches to identify mechanisms that deconvolute whether the microbiome may be a driver of, or offer therapeutic opportunities for, metabolic diseases. Here, we discuss the most promising technological developments for advancing the field.

### Model systems

When comparing model systems for studying the relationship between the GM and metabolic diseases, it is essential to consider both traditional models (e.g. germ-free [GF] and gnotobiotic mice) and emerging technologies (e.g. organs-on-a-chip and nonmurine GF models such as zebrafish and pigs).

GF animals have been used widely to investigate the role of the human GM in obesity and diabetes [1, 173, 175]. These animals, which are born without any microbiota, allow for the interrogation of interventions in the absence of a microbiome. As a result, we can gain insight into whether the microbiome is necessary for a given biological process.

Gnotobiotic disease models are established by colonising GF mice with either an entire GM via donor stool or specific isolated bacterial strains [176]. Studies have demonstrated that GF animals, when inoculated with faecal microbiota from individuals with obesity and type 2 diabetes, successfully replicated disease phenotypes, providing evidence for the involvement of the GM in metabolic diseases [2, 59, 177]. Additionally, an overgrowing endotoxin-producing bacterium, *Enterobacter cloacae* B29, isolated from the gut of a person with morbid obesity and diabetes, induced obesity, fatty liver, and insulin resistance in GF C57BL/6J mice that were otherwise resistant to high-fat diet-induced metabolic defects. Knocking out the endotoxin-producing gene in the B29 bacterial strain or the *Tlr4* gene in C57BL/6J mice prevented the metabolic defects, underscoring the causal relationship between specific gut bacteria and host responses in the initiation and progression of metabolic disease [178–180].

However, certain concepts have been perpetuated about GF mice that are the result of studying only one genotype. For example, GF C57BL/6J mice are resistant to diet-induced obesity [175], whereas GF Swiss Webster mice are not [181]; therefore, because the majority of GF mouse studies use C57BL/6J mice, it has been stated as fact that GF mice in general have to eat more than conventional mice to maintain weight. The divergent responses of these models to high-fat diets underscore the importance of genetic background in research outcomes [182].

The availability of additional GF models, such as pigs and zebrafish, complement the use of GF mice. GF pigs and piglets offer more human-relevant insights than do mice when developing human microbiota-associated gnotobiotic models [183], although the space required to house them is prohibitive for many institutions or limits studies to using just a few animals. GF zebrafish, on the other hand, have proven useful for studies of the GM and distinct host cellular developmental stages [184]. The transparency of the fish body and the ability to fluorescently tag and image different cell types in the presence of different bacteria, as well as the ease of housing and propagating zebrafish, is advantageous for investigating specific questions [185]. These models do not fully replicate human physiology, but they allow longitudinal and invasive sampling in tightly controlled conditions, which is important when asking mechanistic questions.

Organs-on-a-chip, such as the gut-on-a-chip, offer more human-relevant systems because they can be derived directly from human tissue or blood-derived induced pluripotent stem cells, which retain the genetic signature of the host; thus, they enable the study of complex human tissues and cellular interactions in a controlled environment [186]. Recent efforts have demonstrated the ability to seed the gut-on-a-chip with microbiota in a semi-anaerobic environment [187], and many groups are now testing the efficiency of seeding increasingly complex communities on these chips. Although the gut-on-a-chip model lacks some key cell types such as immune cells, major advances include the ability to connect different organ chips such as the gut-chip and neuron-chip [188] to model gut–brain interactions. Creative uses of organs-on-a-chip to study the microbiome will continue to emerge and are likely to fill important gaps to complement animal models.

### Understanding of bacterial genes and functions

The ability to sequence and assemble whole genomes of bacteria is an enormously powerful approach for identifying lineages and the relatedness of bacterial strains and for identifying putative pathways involved in a given bacterial phenotype that may have relevance in human health or disease. If we think about the mechanisms of human disease that have been elucidated from the study of genetically manipulated mice, it is not hard to imagine the wealth of information to be gained from doing the same in bacteria. The ability to knock out and manipulate bacterial genes is not new. Nearly 80 years of bacterial genetics have clarified how pathogens colonise the gut epithelium and secrete toxins, leading to diseases such as cholera, how they share information with each other to adapt to different environments, and how nutritional selection drives their composition in a host. *E. coli* can be considered the bacterial version of the C57BL/6 mouse; its genetics are well-defined and easily engineered [189–191], and it has become the workhorse for testing the effect of modifications in a given environment. However, the commensal GM consists of far more diversity than just *E. coli*; thus, researchers are actively seeking a deeper understanding of GM genetics, using, for example, *Bacteroides* and *Clostridium* as representative organisms [192, 193], as numerous human and mouse studies have demonstrated the important roles of these organisms in health and disease.

Advanced computational tools, including artificial intelligence, have shed new light on unannotated parts of a bacterial genome by predicting the three-dimensional structures of proteins, a task greatly advanced by technologies such as AlphaFold2 [194, 195]. By analysing these structures, researchers can infer possible functions based on their shapes and binding sites. These potential roles can be confirmed by experimental validation in biochemical and microbiological studies [196]. This knowledge, especially regarding how proteins influence metabolic pathways, is crucial for linking microbial activity to health conditions such as diabetes, offering insights into disease mechanisms and potential therapeutic targets.

### Reference-free data analysis

The most critical issue with current database-dependent approaches in microbiome sequencing analysis is their limitation in detecting novel or understudied microbes [197]. When microbial community samples are analysed using databases based on reference genomes from well-characterised bacteria, sequences that do not match are overlooked or misclassified. This process results in a biased view of the microbial ecosystem, potentially missing crucial components that could have significant roles in health and disease, including diabetes. Therefore, advancing microbiome research necessitates the development and use of methods that can uncover and characterise these underrepresented microbial entities.

Assembling genomes de novo from metagenomic sequencing data is a powerful approach in microbiome research that involves constructing genomes directly from sequencing reads without relying on reference databases [197]. This method uses advanced computational algorithms to piece together DNA fragments from a sample, allowing the identification of genetic material from a wide range of organisms, including those not previously sequenced or catalogued. By assembling these genomes, researchers can discover novel species and uncover new gene functions, significantly expanding our understanding of microbial diversity and its potential roles in various environments, including the human body. This approach is particularly useful in revealing the full spectrum of microbial life, including rare or unknown species that might play crucial roles in health and disease.

### Access to the small intestine microbiota

The small intestine is the primary site of nutrient uptake, enterohepatic recycling, and intestinal hormone stimulation; thus, it is essential to gain a deep understanding of microbial function in this region of the body. However, most of our knowledge of human microbiomes has been based on stool samples and the colonic microbiota because accessing the small intestine microbiota is challenging, even with modern endoscopy methods.

Recent advances use innovative methods such as ingestible capsules that sample intestinal material throughout the GI tract [198]. Because each capsule is triggered by a different pH along the gut, this method can provide a microbial atlas of intestinal communities. These tools are being further refined and commercially developed for use in both diagnostics and research. One caveat, however, is that there is potential for microbes to continue growing after the sample has been collected within a capsule, thus giving an inaccurate representation of the native microbiome community. Additionally, these and other capsules have been developed for sampling in the fasting state, leaving the study of postprandial responses still limited, although these responses are likely important to reach a complete understanding of microbial contributions to the regulation of glucose metabolism. Addressing these issues is crucial for ensuring the reliability and accuracy of microbiome studies with such devices.

### Isozyme and small molecule screens

Isozyme and small molecule screens in microbiome research are crucial for identifying specific bacterial products that can be targeted therapeutically. Microbial isozymes are enzymes that have different molecular structures but catalyse the same reaction as the host enzymes. Screening these products can reveal variations in microbial metabolism that might influence health and potentially interfere with medications, as in the case of bacterial DPP-4 isozymes [33]. Small molecule screens focus on identifying bioactive compounds produced by microbes [199]. These compounds can have significant effects on host pathophysiology [140, 200]. By identifying specific isozymes and small molecules, researchers can target them for degradation or enhancement, offering potential therapeutic strategies for diseases such as diabetes.

## Potential GM-based diagnostics and therapies in diabetes

As described above, no diagnostic and generalised faecal microbiota taxonomic signature has been found for type 1 or type 2 diabetes [86, 201]. Future research should therefore move toward strain-level studies in large prospective populations and, when possible, focus on functional profiling of intestinal microbes along the GI tract [198], with special attention to stable isotope precursors to study production and substrate fluxes of important microbially produced metabolites in different GI regions [202].

### High-fibre diets and SCFA-based treatments

With regard to GM-based therapies for diabetes, high-fibre diets have been shown to be effective in controlling blood glucose levels and reducing insulin resistance in both type 1 and type 2 diabetes [203, 204]. Although the direct mode of action of dietary fibre via the GM remains to be shown, these trials underscore the potential importance of including GM modulation strategies as part of diabetes intervention trials, especially for the production of beneficial metabolites such as SCFAs [205]. However, as noted above, intervention trials of oral SCFA butyrate supplementation have shown no effect on glycaemic control or other markers of diabetes regulation in either type 1 or type 2 diabetes [116, 122, 123, 206], probably because the site of delivery does not mimic endogenous production. For other SCFAs, including propionate and acetate, data are too scarce to draw any conclusions regarding possible effects on metabolic regulation.

### Conventional and next-generation probiotics

Probiotic therapies for diabetes can be divided into conventional probiotics, particularly *Lactobacillus* and *Bifidobacterium* strains, which have a history of use for human consumption in fermented foods or supplements to promote health, and next-generation probiotics, which are strains of new bacterial species recently identified as indigenous members of the human GM. These strains are associated with health, and their presence is diminished in disease settings [76, 207]. With regard to the conventional probiotics, prospective randomised controlled trials (RCTs) in new-onset type 1 diabetes are ongoing (NCT03961854, NCT03961347, NCT04769037, and NCT05767450), and a smaller trial has shown only moderate effects in longstanding type 1 diabetes [208]. However, an open-label trial of probiotics (strains of *Bifidobacterium*, *Lactobacillus*, and *Streptococcus salivarius*) found beneficial effects on susceptibility to and progression of type 1 diabetes in siblings of people with type 1 diabetes [209]. In type 2 diabetes, a recent meta-analysis described some efficacy of these probiotic strains in metabolic control and reduced insulin resistance [210].

With regard to next-generation probiotics, fewer data have been generated in humans. For example, despite specific strains (e.g. *Akkermansia*) having been associated with a healthy metabolic phenotype [211], an RCT intervention with *A. muciniphila* did not identify strong metabolic effects [143]. This finding could be the result of a lack of a causal role of these tested strains in the metabolic syndrome, reduced viability upon passage through the stomach [212, 213], inadequate dosages, or a lack of engraftment when introduced in the human gut [214]. Because the small intestine is important for the pathophysiology of both type 1 and type 2 diabetes, further analyses of small intestinal microbiota from individuals with type 1 [215] and type 2 [216, 217] diabetes are needed, with defined combinations of next-generation probiotic strains studied as possible interventions for diabetes. However, this effort should consider conditions of bacterial strain engraftment, ecological or functional dependencies on other bacterial members, and potential redundancies in functionality, as shown by a meta-analysis of FMT [218].

### Donor FMT

Until such investigations with defined combinations of strains are completed, donor FMT might provide insight into the magnitude of effect of modulating the GM and the effect of such modulation on the pathophysiology and potential reversibility of diabetes. de Groot et al [219] recently published research on the efficacy of fresh FMT in maintaining residual beta cell function and dampening autoimmunity in new-onset type 1 diabetes. Other studies have been performed for type 2 diabetes and insulin resistance, showing a modest effect of FMT on insulin resistance and non-alcoholic fatty liver disease [216, 217, 220–223], whereas one study showed no effect on these parameters [224]. Additionally, a combined intervention of encapsulated donor FMT and fibre supplementation showed beneficial effects on glucose metabolism, suggesting the possible need to design interventions not only with synthetic bacterial strain consortia, but also with dietary support (e.g. fibre to nourish the bacterial strains) [221]. Finally, studies evaluating whether autologous FMT after lifestyle intervention could help prevent weight regain have suggested that diet-induced changes in low-abundance bacteria might be responsible for weight loss maintenance, which could guide more precise interventions with less ethical burden and lower risks of transmitting diseases [225].

Overall, donor FMT is a more diffuse approach than interventions with targeted strains or metabolites [226]. Additionally, there are differences in mode of faecal material administration (capsules vs fresh FMT), intestinal pH (e.g. due to antacids), and colonic transit time in existing datasets, and the amount of faecal microbiota administered also seems to affect engraftment of donor bacterial strains [218, 227]. These factors preclude generalisation of the results of studies to date. We therefore advocate further standardisation of intestinal microbiota composition measurements [228], with strict dietary monitoring. Also, better standardisation is needed in human studies of FMT-based interventions. In this context, production of lyophilised capsules for FMT must follow Good Manufacturing Practices to maintain viability and ensure adequate shelf life [229].

Nevertheless, based on its wide availability and general safety (provided that donors are adequately screened [230]), FMT could provide clinicians with new treatment modalities for diabetes until interventions with defined combinations of strains are available, especially if next-generation probiotics can be spiked in donor faecal microbiota to boost therapeutic efficacy [231]. However, these interventions should adhere to the international Nagoya Protocol on Access to Genetic Resources and the Fair and Equitable Sharing of Benefits Arising From Their Utilization to the Convention on Biological Diversity [232], which seeks to prevent researchers or their institutions from financially capitalising (at the expense of vulnerable individuals or populations) on identified bacterial strains as next-generation probiotics. With regard to trial outcomes for diabetes and GM-based therapies, using dynamic measurements of glucose metabolism over time (e.g. mixed-meal tests or continuous glucose monitoring) could provide better insights into the interactions between the GM, diet, and glucose homeostasis during both FMT and administration of defined strain combinations.

New insights into the GM are increasingly associating it with diabetes in humans, although the microbiome of the small intestine remains understudied. Intervention studies with FMT in humans have been able to dissect associations from causality and have indeed shown some clinical benefit, although the contrast between, on average, relatively small therapeutic effects and ethical concerns [233] preclude widespread practical use of this treatment option in diabetes clinical care. Additional studies are thus needed of prospective associations between the GM and diabetes in multiethnic cohorts. Alongside this effort, the therapeutic potential of synthetic GM-derived bacterial strains and/or communities and engineered systems for targeting intestinal delivery of identified metabolites in diabetes should be explored.

## Conclusion

Over the past two decades, alterations in the GM have been associated with aberrant glucose metabolism and steatosis in individuals with diabetes. Larger sample sizes in epidemiological studies have now started to show the magnitude and possible consistency of correlations between the GM and human metabolic traits of relevance to obesity and/or type 2 diabetes; however, for type 1 diabetes, the picture is much less clear.

Interaction with diabetes medications in relation to ethnicity and dietary intake should be taken into account more rigorously in future studies. Moreover, in recent years, more insights have been gained into the function of the GM beyond just its composition, and this information nicely dovetails with earlier reports of links between specific metabolites, including SCFAs, BCAAs and bile acids, and obesity and diabetes.

With regard to GM composition, only a few studies have addressed the role of phages and fungi and the interactions between these inhabitants and bacterial strains in diabetes. It is clear that future studies also need to focus on small intestine microbiota function, as well as developing adequate bioinformatic pipelines and correctly assembling genomes (see textbox).

We must also take into account that most data to date have been generated in mouse studies, whose relevance to human diabetes needs further confirmation because of the large differences between mice and humans in diet, genetics, and life span. Nevertheless, human intervention studies of single strains and FMT in the setting of human diabetes have shown a range of clinical metabolic effects (compared with the more consistent effects of medications), yet without serious side effects. In conclusion, after almost two decades of study, we must still look to future efforts to illuminate the clinical diagnostic and therapeutic applicability of GM research to human diabetes.

## Supplementary Information

Below is the link to the electronic supplementary material.

Supplementary file1 (PPTX 746 KB)

## Abbreviations

AHR: Aryl hydrocarbon receptor
BCAA: Branched-chain amino acid
BMI: Body mass index
DPP-4: Dipeptidyl peptidase 4
EEC: Enteroendocrine cell
FMT: Faecal microbiota transplantation
FXR: Farnesoid X receptor
GF: Germ-free
GI: Gastrointestinal
GLP-1: Glucagon-like peptide 1
GM: Gut microbiota
GPCR: G-protein-coupled receptor
LPS: Lipopolysaccharide
MR: Mendelian randomisation
PAMP: Pathogen-associated molecular pattern
RCT: Randomised controlled trial
SCFA: Short-chain fatty acid
SGLT2: Sodium–glucose cotransporter 2
TGR5: Takeda GPCR 5
TLR4: Toll-like receptor 4
TMAO: Trimethylamine oxide

## Authors’ relationships and activities

JHN has served as an advisor for Abbvie, Bristol Myers Squibb, Falk, Janssen, Roche, and Takeda. MN is the founder and a scientific advisory board member of Caelus Pharmaceuticals and Advanced Microbiome Interventions in Amsterdam, the Netherlands. YS is co-author of patents based on the use of intestinal bacteria for obesity and metabolic comorbidities. VT holds a patent for the use of microbiota in type 2 diabetes. LZ is a co-founder of Notitia Biotechnologies, a microbiome company providing products related to diabetes. All other authors declare that there are no relationships or activities that might bias, or be perceived to bias, their work.

## References

[1] Bäckhed F, Ding H, Wang T et al (2004) The gut microbiota as an environmental factor that regulates fat storage. Proc Natl Acad Sci U S A 101:15718–15723. 10.1073/pnas.0407076101 15505215 10.1073/pnas.0407076101 PMC524219

[2] Ridaura VK, Faith JJ, Rey FE et al (2013) Gut microbiota from twins discordant for obesity modulate metabolism in mice. Science 341:1241214. 10.1126/science.1241214 24009397 10.1126/science.1241214 PMC3829625

[3] Sze MA, Schloss PD (2016) Looking for a signal in the noise: revisiting obesity and the microbiome. mBio 7:e01018-16. 10.1128/mBio.01018-16 27555308 10.1128/mBio.01018-16 PMC4999546

[4] Duvallet C, Gibbons SM, Gurry T, Irizarry RA, Alm EJ (2017) Meta-analysis of gut microbiome studies identifies disease-specific and shared responses. Nat Commun 8:1784. 10.1038/s41467-017-01973-8 29209090 10.1038/s41467-017-01973-8 PMC5716994

[5] Pasolli E, Truong DT, Malik F, Waldron L, Segata N (2016) Machine learning meta-analysis of large metagenomic datasets: tools and biological insights. PLoS Comput Biol 12:e1004977. 10.1371/journal.pcbi.1004977 27400279 10.1371/journal.pcbi.1004977 PMC4939962

[6] Rothschild D, Leviatan S, Hanemann A, Cohen Y, Weissbrod O (2022) An atlas of robust microbiome associations with phenotypic traits based on large-scale cohorts from two continents. PLoS One 17:e0265756. 10.1371/journal.pone.0265756 35324954 10.1371/journal.pone.0265756 PMC8947124

[7] Qin J, Li Y, Cai Z et al (2012) A metagenome-wide association study of gut microbiota in type 2 diabetes. Nature 490:55–60. 10.1038/nature11450 23023125 10.1038/nature11450

[8] Le Chatelier E, Nielsen T, Qin J et al (2013) Richness of human gut microbiome correlates with metabolic markers. Nature 500:541–546. 10.1038/nature12506 23985870 10.1038/nature12506

[9] Allin KH, Tremaroli V, Caesar R et al (2018) Aberrant intestinal microbiota in individuals with prediabetes. Diabetologia 61:810–820. 10.1007/s00125-018-4550-1 29379988 10.1007/s00125-018-4550-1 PMC6448993

[10] Forslund K, Hildebrand F, Nielsen T et al (2015) Disentangling type 2 diabetes and metformin treatment signatures in the human gut microbiota. Nature 528:262–266. 10.1038/nature15766 26633628 10.1038/nature15766 PMC4681099

[11] Karlsson FH, Tremaroli V, Nookaew I et al (2013) Gut metagenome in European women with normal, impaired and diabetic glucose control. Nature 498:99–103. 10.1038/nature12198 23719380 10.1038/nature12198

[12] Wu H, Tremaroli V, Schmidt C et al (2020) The gut microbiota in prediabetes and diabetes: a population-based cross-sectional study. Cell Metab 32:379-390.e3. 10.1016/j.cmet.2020.06.011 32652044 10.1016/j.cmet.2020.06.011

[13] Alvarez-Silva C, Kashani A, Hansen TH et al (2021) Trans-ethnic gut microbiota signatures of type 2 diabetes in Denmark and India. Genome Med 13:37. 10.1186/s13073-021-00856-4 33658058 10.1186/s13073-021-00856-4 PMC7931542

[14] Zhou W, Sailani MR, Contrepois K et al (2019) Longitudinal multi-omics of host-microbe dynamics in prediabetes. Nature 569:663–671. 10.1038/s41586-019-1236-x 31142858 10.1038/s41586-019-1236-x PMC6666404

[15] Sayols-Baixeras S, Dekkers KF, Baldanzi G et al (2023) *Streptococcus* species abundance in the gut is linked to subclinical coronary atherosclerosis in 8973 participants from the SCAPIS cohort. Circulation 148:459–472. 10.1161/CIRCULATIONAHA.123.063914 37435755 10.1161/CIRCULATIONAHA.123.063914 PMC10399955

[16] Grahnemo L, Nethander M, Coward E et al (2022) Cross-sectional associations between the gut microbe Ruminococcus gnavus and features of the metabolic syndrome. Lancet Diabetes Endocrinol 10:481–483. 10.1016/S2213-8587(22)00113-9 35662399 10.1016/S2213-8587(22)00113-9

[17] Belda E, Voland L, Tremaroli V et al (2022) Impairment of gut microbial biotin metabolism and host biotin status in severe obesity: effect of biotin and prebiotic supplementation on improved metabolism. Gut 71:2463–2480. 10.1136/gutjnl-2021-325753 35017197 10.1136/gutjnl-2021-325753 PMC9664128

[18] Pedersen HK, Gudmundsdottir V, Nielsen HB et al (2016) Human gut microbes impact host serum metabolome and insulin sensitivity. Nature 535:376–381. 10.1038/nature18646 27409811 10.1038/nature18646

[19] Wang TJ, Larson MG, Vasan RS et al (2011) Metabolite profiles and the risk of developing diabetes. Nat Med 17:448–453. 10.1038/nm.2307 21423183 10.1038/nm.2307 PMC3126616

[20] Mueller NT, Differding MK, Zhang M et al (2021) Metformin affects gut microbiome composition and function and circulating short-chain fatty acids: a randomized trial. Diabetes Care 44:1462–1471. 10.2337/dc20-2257 34006565 10.2337/dc20-2257 PMC8323185

[21] Wu H, Esteve E, Tremaroli V et al (2017) Metformin alters the gut microbiome of individuals with treatment-naive type 2 diabetes, contributing to the therapeutic effects of the drug. Nat Med 23:850–858. 10.1038/nm.4345 28530702 10.1038/nm.4345

[22] Pryor R, Norvaisas P, Marinos G et al (2019) Host-microbe-drug-nutrient screen identifies bacterial effectors of metformin therapy. Cell 178:1299-1312.e29. 10.1016/j.cell.2019.08.003 31474368 10.1016/j.cell.2019.08.003 PMC6736778

[23] Dekkers KF, Sayois-Baixeras S, Baldanzi G et al (2022) An online atlas of human plasma metabolite signatures of gut microbiome composition. Nat Commun 13:5370. 10.1038/s41467-022-33050-0 36151114 10.1038/s41467-022-33050-0 PMC9508139

[24] Sun L, Xie C, Wang G et al (2018) Gut microbiota and intestinal FXR mediate the clinical benefits of metformin. Nat Med 24:1919–1929. 10.1038/s41591-018-0222-4 30397356 10.1038/s41591-018-0222-4 PMC6479226

[25] Bryrup T, Thomsen CW, Kern T et al (2019) Metformin-induced changes of the gut microbiota in healthy young men: results of a non-blinded, one-armed intervention study. Diabetologia 62:1024–1035. 10.1007/s00125-019-4848-7 30904939 10.1007/s00125-019-4848-7 PMC6509092

[26] Gu Y, Wang X, Li J et al (2017) Analyses of gut microbiota and plasma bile acids enable stratification of patients for antidiabetic treatment. Nat Commun 8:1785. 10.1038/s41467-017-01682-2 29176714 10.1038/s41467-017-01682-2 PMC5702614

[27] van Bommel EJM, Herrema H, Davids M, Kramer MHH, Nieuwdorp M, van Raaite DH (2020) Effects of 12-week treatment with dapagliflozin and gliclazide on faecal microbiome: results of a double-blind randomized trial in patients with type 2 diabetes. Diabetes Metab 46:164–168. 10.1016/j.diabet.2019.11.005 31816432 10.1016/j.diabet.2019.11.005

[28] Smits MM, Fluitman KS, Herrema H et al (2021) Liraglutide and sitagliptin have no effect on intestinal microbiota composition: a 12-week randomized placebo-controlled trial in adults with type 2 diabetes. Diabetes Metab 47:101223. 10.1016/j.diabet.2021.101223 33429063 10.1016/j.diabet.2021.101223

[29] Bica I-C, Pietroșel VA, Salmen T et al (2023) The effects of cardioprotective antidiabetic therapy on microbiota in patients with type 2 diabetes mellitus: a systematic review. Int J Mol Sci 24:7184. 10.3390/ijms24087184 37108347 10.3390/ijms24087184 PMC10138454

[30] Moon JS, Hong JH, Jung YJ, Ferrannini E, Nauck MA, Lim S (2022) SGLT-2 inhibitors and GLP-1 receptor agonists in metabolic dysfunction-associated fatty liver disease. Trends Endocrinol Metab 33:424–442. 10.1016/j.tem.2022.03.005 35491295 10.1016/j.tem.2022.03.005

[31] Wong CK, Yusta B, Koehler JA et al (2022) Divergent roles for the gut intraepithelial lymphocyte GLP-1R in control of metabolism, microbiota, and T cell-induced inflammation. Cell Metab 34:1514-1531.e7. 10.1016/j.cmet.2022.08.003 36027914 10.1016/j.cmet.2022.08.003

[32] Palacios T, Vitetta L, Coulson S et al (2020) Targeting the intestinal microbiota to prevent type 2 diabetes and enhance the effect of metformin on glycaemia: a randomized controlled pilot study. Nutrients 12:2041. 10.3390/nu12072041 32660025 10.3390/nu12072041 PMC7400852

[33] Wang K, Zhang Z, Hang J et al (2023) Microbial-host-isozyme analyses reveal microbial DPP4 as a potential antidiabetic target. Science 381:eadd5787. 10.1126/science.add5787 37535747 10.1126/science.add5787

[34] Olivare M, Schüppel V, Hassan AM et al (2018) The potentialrole of the dipeptidyl peptidase-4-like activity from the gut microbiota on the host health. Front Microbiol 9:1900. 10.3389/fmicb.2018.01900 30186247 10.3389/fmicb.2018.01900 PMC6113382

[35] Boets E, Gomand SV, Deroover L et al (2017) Systemic availability and metabolism of colonic-derived short-chain fatty acids in healthy subjects: a stable isotope study. J Physiol 595:541–555. 10.1113/JP272613 27510655 10.1113/JP272613 PMC5233652

[36] Morrison DJ, Preston T (2016) Formation of short chain fatty acids by the gut microbiota and their impact on human metabolism. Gut Microbes 7:189–200. 10.1080/19490976.2015.1134082 26963409 10.1080/19490976.2015.1134082 PMC4939913

[37] Sanna S, van Zuydam NR, Mahajan A et al (2019) Causal relationships among the gut microbiome, short-chain fatty acids and metabolic diseases. Nat Genet 51:600–605. 10.1038/s41588-019-0350-x 30778224 10.1038/s41588-019-0350-x PMC6441384

[38] Chávez-Talavera O, Haas J, Grzych G, Tailleux A, Staels B (2019) Bile acid alterations in nonalcoholic fatty liver disease, obesity, insulin resistance and type 2 diabetes: what do the human studies tell? Curr Opin Lipidol 30:244–254. 10.1097/MOL.0000000000000597 30893108 10.1097/MOL.0000000000000597

[39] Vincent RP, Omar S, Ghozlan S et al (2013) Higher circulating bile acid concentrations in obese patients with type 2 diabetes. Ann Clin Biochem 50:360–364. 10.1177/0004563212473450 23771134 10.1177/0004563212473450

[40] Prawitt J, Caron S, Staels B (2011) Bile acid metabolism and the pathogenesis of type 2 diabetes. Curr Diab Rep 11:160–166. 10.1007/s11892-011-0187-x 21431855 10.1007/s11892-011-0187-x PMC3338411

[41] Haeusler RA, Astiarraga B, Camastra S, Accili D, Ferrannini E (2013) Human insulin resistance is associated with increased plasma levels of 12α-hydroxylated bile acids. Diabetes 62:4184–4191. 10.2337/db13-0639 23884887 10.2337/db13-0639 PMC3837033

[42] Zheng X, Chen T, Zhao A et al (2021) Hyocholic acid species as novel biomarkers for metabolic disorders. Nat Commun 12:1487. 10.1038/s41467-021-21744-w 33674561 10.1038/s41467-021-21744-w PMC7935989

[43] Petersen AØ, Julienne H, Hyötyläinen T et al (2021) Conjugated C-6 hydroxylated bile acids in serum relate to human metabolic health and gut Clostridia species. Sci Rep 11:13252. 10.1038/s41598-021-91482-y 34168163 10.1038/s41598-021-91482-y PMC8225906

[44] Chen L, van den Munckhof CL, Schraa K et al (2020) Genetic and microbial associations to plasma and fecal bile acids in obesity relate to plasma lipids and liver fat content. Cell Rep 33:108212. 10.1016/j.celrep.2020.108212 33027657 10.1016/j.celrep.2020.108212

[45] Browning MG, Pessoa BM, Khoraki J, Campos GM (2019) Changes in bile acid metabolism, transport, and signaling as central drivers for metabolic improvements after bariatric surgery. Curr Obes Rep 8:175–184. 10.1007/s13679-019-00334-4 30847736 10.1007/s13679-019-00334-4

[46] van den Broek M, de Heide LJM, Sips FLP et al (2021) Altered bile acid kinetics contribute to postprandial hypoglycaemia after Roux-en-Y gastric bypass surgery. Int J Obes (Lond) 45:619–630. 10.1038/s41366-020-00726-w 33452416 10.1038/s41366-020-00726-w PMC7906904

[47] de Mello VD, Paananen J, Lindström J et al (2017) Indolepropionic acid and novel lipid metabolites are associated with a lower risk of type 2 diabetes in the Finnish Diabetes Prevention Study. Sci Rep 7:46337. 10.1038/srep46337 28397877 10.1038/srep46337 PMC5387722

[48] Molinaro A, Lassen PB, Henricsson M et al (2020) Imidazole propionate is increased in diabetes and associated with dietary patterns and altered microbial ecology. Nat Commun 11:5881. 10.1038/s41467-020-19589-w 33208748 10.1038/s41467-020-19589-w PMC7676231

[49] Nemet I, Li XS, Haghikia A et al (2023) Atlas of gut microbe-derived products from aromatic amino acids and risk of cardiovascular morbidity and mortality. Eur Heart J 44:3085–3096. 10.1093/eurheartj/ehad333 37342006 10.1093/eurheartj/ehad333 PMC10481777

[50] Molinaro A, Nemet I, Lassen PB et al (2023) Microbially produced imidazole propionate is associated with heart failure and mortality. JACC Heart Fail 11:810–821. 10.1016/j.jchf.2023.03.008 37115134 10.1016/j.jchf.2023.03.008 PMC12512386

[51] Meijnikman A, Davids M, Herrema H et al (2022) Microbiome-derived ethanol in nonalcoholic fatty liver disease. Nat Med 28:2100–2106. 10.1038/s41591-022-02016-6 36216942 10.1038/s41591-022-02016-6

[52] Yuan J, Chen C, Cui J et al (2019) Fatty liver disease caused by high-alcohol-producing Klebsiella pneumoniae. Cell Metab 30:675-688.e7. 10.1016/j.cmet.2019.08.018 31543403 10.1016/j.cmet.2019.08.018

[53] Nair S, Cope K, Risby TH, Diehl AM (2001) Obesity and female gender increase breath ethanol concentration: potential implications for the pathogenesis of nonalcoholic steatohepatitis. Am J Gastroenterol 96:1200–1204. 10.1111/j.1572-0241.2001.03702.x 11316170 10.1111/j.1572-0241.2001.03702.x

[54] Jia J, Dou P, Gao M et al (2019) Assessment of causal direction between gut microbiota-dependent metabolites and cardiometabolic health: a bidirectional Mendelian randomization analysis. Diabetes 68:1747–1755. 10.2337/db19-0153 31167879 10.2337/db19-0153

[55] Li H, Li C (2023) Causal relationship between gut microbiota and type 2 diabetes: a two-sample Mendelian randomization study. Front Microbiol 14:1184734. 10.3389/fmicb.2023.1184734 37692402 10.3389/fmicb.2023.1184734 PMC10483233

[56] Diabetes and Nutrition Study Group (DNSG) of the European Association for the Study of Diabetes (2023) Evidence-based European recommendations for the dietary management of diabetes. Diabetologia 66:965–985. 10.1007/s00125-023-05894-8 37069434 10.1007/s00125-023-05894-8

[57] Makki K, Deehan EC, Walter J, Backhed F (2018) The impact of dietary fiber on gut microbiota in host health and disease. Cell Host Microbe 23:705–715. 10.1016/j.chom.2018.05.012 29902436 10.1016/j.chom.2018.05.012

[58] Kovatcheva-Datchary P, Nilsson A, Akrami R et al (2015) Dietary fiber-induced improvement in glucose metabolism is associated with increased abundance of Prevotella. Cell Metab 22:971–982. 10.1016/j.cmet.2015.10.001 26552345 10.1016/j.cmet.2015.10.001

[59] Zhao L, Zhang F, Ding X et al (2018) Gut bacteria selectively promoted by dietary fibers alleviate type 2 diabetes. Science 359:1151–1156. 10.1126/science.aao5774 29590046 10.1126/science.aao5774

[60] Gardner CD, Trepanowski JF, Del Gobbo LC et al (2018) Effect of low-fat vs low-carbohydrate diet on 12-month weight loss in overweight adults and the association with genotype pattern or insulin secretion: the DIETFITS randomized clinical trial. JAMA 319:667–679. 10.1001/jama.2018.0245 29466592 10.1001/jama.2018.0245 PMC5839290

[61] Guthrie L, Spencer SP, Perelman D et al (2022) Impact of a 7-day homogeneous diet on interpersonal variation in human gut microbiomes and metabolomes. Cell Host Microbe 30:863-874.e4. 10.1016/j.chom.2022.05.003 35643079 10.1016/j.chom.2022.05.003 PMC9296065

[62] Ordovas JM, Ferguson LR, Tai ES, Mathers JC (2018) Personalised nutrition and health. BMJ 361:bmj.k2173. 10.1136/bmj.k2173 29898881 10.1136/bmj.k2173 PMC6081996

[63] Zeevi D, Korem T, Zmora N et al (2015) Personalized nutrition by prediction of glycemic responses. Cell 163:1079–1094. 10.1016/j.cell.2015.11.001 26590418 10.1016/j.cell.2015.11.001

[64] Berry SE, Valdes AM, Drew DA et al (2020) Human postprandial responses to food and potential for precision nutrition. Nat Med 26:964–973. 10.1038/s41591-020-0934-0 32528151 10.1038/s41591-020-0934-0 PMC8265154

[65] Almeida A, Nayfach S, Boland M et al (2021) A unified catelog of 204,938 reference genomes from the human gut microbiome. Nat Biotechnol 39:105–114. 10.1038/s41587-020-0603-3 32690973 10.1038/s41587-020-0603-3 PMC7801254

[66] Zeng S, Patangia D, Almeida A et al (2022) A compendium of 32,277 metagenome-assembled genomes and over 80 mission genes from the early-life human gut microbiome. Nat Commun 13:5139. 10.1038/s41467-022-32805-z 36050292 10.1038/s41467-022-32805-z PMC9437082

[67] Leviatan S, Shoer S, Rothschild D, Gorodetski M, Segal E (2022) An expanded reference map of the human gut microbiome reveals hundreds of previously unknown species. Nat Commun 13:3863. 10.1038/s41467-022-31502-1 35790781 10.1038/s41467-022-31502-1 PMC9256738

[68] Tsilimigras MC, Fodor AA (2016) Compositional data analysis of the microbiome: fundamentals, tools, and challenges. Ann Epidemiol 26:330–335. 10.1016/j.annepidem.2016.03.002 27255738 10.1016/j.annepidem.2016.03.002

[69] Olsson LM, Boulund F, Nilsson S et al (2022) Dynamics of the normal gut microbiota: a longitudinal one-year population study in Sweden. Cell Host Microbe 30:726-739.e3. 10.1016/j.chom.2022.03.002 35349787 10.1016/j.chom.2022.03.002

[70] Chen L, Wang D, Garmaeva S et al (2021) The long-term genetic stability and individual specificiaty of the human gut microbiome. Cell 184:2302-2315.e12. 10.1016/j.cell.2021.03.024 33838112 10.1016/j.cell.2021.03.024

[71] Abdellaoui A, Yengo L, Verweij KJH, Visscher PM (2023) 15 years of GWAS discovery: realizing the promise. Am J Hum Genet 110:179–194. 10.1016/j.ajhg.2022.12.011 36634672 10.1016/j.ajhg.2022.12.011 PMC9943775

[72] Kullo IJ, Lewis CM, Inouye M, Martin AR, Ripatti S, Chatterjee N (2022) Polygenic scores in biomedical research. Nat Rev Genet 23:524–532. 10.1038/s41576-022-00470-z 35354965 10.1038/s41576-022-00470-z PMC9391275

[73] Wang H, Gou W, Su C et al (2022) Association of gut microbiota with glycaemic traits and incident type 2 diabetes, and modulation by habitual diet: a population-based longitudinal cohort study in Chinese adults. Diabetologia 65:1145–1156. 10.1007/s00125-022-05687-5 35357559 10.1007/s00125-022-05687-5 PMC9174105

[74] Vals-Delgado C, Alcala-Diaz JF, Molina-Abril H et al (2021) An altered microbiota pattern preceded type 2 diabetes mellitus development: from the CORDIOPREV study. J Adv Res 35:99–108. 10.1016/j.jare.2021.05.001 35024196 10.1016/j.jare.2021.05.001 PMC8721255

[75] Ruuskanen MO, Erawijantari PP, Havulinna AS et al (2022) Gut microbiome composition is predictive of incident type 2 diabetes in a population cohort of 5,572 Finnish adults. Diabetes Care 45:811–818. 10.2337/dc21-2358 35100347 10.2337/dc21-2358 PMC9016732

[76] De Filippis F, Esposito A, Ercolini D (2022) Outlook on next-generation probiotics from the human gut. Cell Mol Life Sci 79:76. 10.1007/s00018-021-04080-6 35043293 10.1007/s00018-021-04080-6 PMC11073307

[77] De Filippis F, Pasolli E, Ercolini D (2020) Newly explored Faecalibacterium diversity is connected to age, lifestyle, geography, and disease. Curr Biol 30:4932-4943.e4. 10.1016/j.cub.2020.09.063 33065016 10.1016/j.cub.2020.09.063

[78] Tett A, Huang KD, Asnicar F et al (2019) The Prevotella copri complex comprises four distinct clades underrepresented in westernized populations. Cell Host Microbe 26:666-679.e7. 10.1016/j.chom.2019.08.018 31607556 10.1016/j.chom.2019.08.018 PMC6854460

[79] Blanco-Miguez A, Gálvez EJC, Pasolli E et al (2023) Extension of the Segatella copri complex to 13 species with distinct large extrachromosomal elements and associations with host conditions. Cell Host Microbe 31:1804-1819.e9. 10.1016/j.chom.2023.09.013 37883976 10.1016/j.chom.2023.09.013 PMC10635906

[80] Van Rossum T, Ferretti P, Maistrenko OM, Bork P (2020) Diversity within species: interpreting strains in microbiomes. Nat Rev Microbiol 18:491–506. 10.1038/s41579-020-0368-1 32499497 10.1038/s41579-020-0368-1 PMC7610499

[81] Ma Y, You X, Mai G, Tokuyasu T, Liu C (2018) A human gut phage catalog correlates the gut phageome with type 2 diabetes. Microbiome 6:24. 10.1186/s40168-018-0410-y 29391057 10.1186/s40168-018-0410-y PMC5796561

[82] Yang K, Niu J, Zuo T et al (2021) Alterations in the gut virome in obesity and type 2 diabetes mellitus. Gastroenterology 161:1257-1269.e13. 10.1053/j.gastro.2021.06.056 34175280 10.1053/j.gastro.2021.06.056

[83] de Jonge PA, Wortelboer K, Scheithauer TPM et al (2022) Gut virome profiling identifies a widespread bacteriophage family associated with metabolic syndrome. Nat Commun 13:3594. 10.1038/s41467-022-31390-5 35739117 10.1038/s41467-022-31390-5 PMC9226167

[84] Lawrence D, Baldridge MT, Handley SA (2019) Phages and human health: more than idle hitchhikers. Viruses 11:587. 10.3390/v11070587 31252683 10.3390/v11070587 PMC6669647

[85] Garmaeva S, Sinha T, Kurilshikov A, Fu J, Wijmenga C, Zhernakova A (2019) Studying the gut virome in the metagenomic era: challenges and perspectives. BMC Biol 17:84. 10.1186/s12915-019-0704-y 31660953 10.1186/s12915-019-0704-y PMC6819614

[86] Talmor-Barkan Y, Bar N, Shaul AA et al (2022) Metabolomic and microbiome profiling reveals personalized risk factors for coronary artery disease. Nat Med 28:295–302. 10.1038/s41591-022-01686-6 35177859 10.1038/s41591-022-01686-6 PMC12365913

[87] Fromentin S, Forslund SK, Chechi K et al (2022) Microbiome and metabolome features of the cardiometabolic disease spectrum. Nat Med 28:303–314. 10.1038/s41591-022-01688-4 35177860 10.1038/s41591-022-01688-4 PMC8863577

[88] Asnicar F, Berry SE, Valdes AM et al (2021) Microbiome connections with host metabolism and habitual diet from 1,098 deeply phenotyped individuals. Nat Med 27:321–332. 10.1038/s41591-020-01183-8 33432175 10.1038/s41591-020-01183-8 PMC8353542

[89] Hug LA, Baker BJ, Anantharaman K et al (2016) A new view of the tree of life. Nat Microbiol 1:16048. 10.1038/nmicrobiol.2016.48 27572647 10.1038/nmicrobiol.2016.48

[90] Bogaert D, van Beveren GJ, de Koff EM et al (2023) Mother-to-infant microbiota transmission and infant microbiota development across multiple body sites. Cell Host Microbe 31:447-460.e6. 10.1016/j.chom.2023.01.018 36893737 10.1016/j.chom.2023.01.018

[91] Dominguez-Bello MG, De Jesus-Laboy KM, Shen N et al (2016) Partial restoration of the microbiota of cesarean-born infants via vaginal microbial transfer. Nat Med 22:250–253. 10.1038/nm.4039 26828196 10.1038/nm.4039 PMC5062956

[92] Cox LM, Yamanishi S, Sohn J et al (2014) Altering the intestinal microbiota during a critical developmental window has lasting metabolic consequences. Cell 158:705–721. 10.1016/j.cell.2014.05.052 25126780 10.1016/j.cell.2014.05.052 PMC4134513

[93] Falony G, Joossens M, Vieira-Silva S et al (2016) Population-level analysis of gut microbiome variation. Science 352:560–564. 10.1126/science.aad3503 27126039 10.1126/science.aad3503

[94] Rollenske T, Burkhalter S, Muerner L et al (2021) Parallelism of intestinal secretory IgA shapes functional microbial fitness. Nature 598:657–661. 10.1038/s41586-021-03973-7 34646015 10.1038/s41586-021-03973-7

[95] Kabbert J, Benckert J, Rollenske T et al (2020) High microbiota reactivity of adult human intestinal IgA requires somatic mutations. J Exp Med 217:e20200275. 10.1084/jem.20200275 32640466 10.1084/jem.20200275 PMC7526496

[96] Li H, Limenitakis JP, Greiff V et al (2020) Mucosal or systemic microbiota exposures shape the B cell repertoire. Nature 584:274–278. 10.1038/s41586-020-2564-6 32760003 10.1038/s41586-020-2564-6

[97] Nyström EEL, Martinez-Abad B, Arike L et al (2021) An intercrypt subpopulation of goblet cells is essential for colonic mucus barrier function. Science 372:eabb1590. 10.1126/science.abb1590 33859001 10.1126/science.abb1590 PMC8542866

[98] Li T, Ding N, Guo H et al (2024) A gut microbiota-bile acid axis promotes intestinal homeostasis upon aspirin-mediated damage. Cell Host Microbe 32:191-208.e9. 10.1016/j.chom.2023.12.015 38237593 10.1016/j.chom.2023.12.015 PMC10922796

[99] Spadoni I, Zagato E, Bertocchi A et al (2015) A gut-vascular barrier controls the systemic dissemination of bacteria. Science 350:830–834. 10.1126/science.aad0135 26564856 10.1126/science.aad0135

[100] Macpherson AJ, Uhr T (2004) Induction of protective IgA by intestinal dendritic cells carrying commensal bacteria. Science 303:1662–1665. 10.1126/science.1091334 15016999 10.1126/science.1091334

[101] Balmer ML, Slack E, de Gottardi A et al (2014) The liver may act as a firewall mediating mutualism between the host and its gut commensal microbiota. Sci Transl Med 6:237ra66. 10.1126/scitranslmed.3008618 24848256 10.1126/scitranslmed.3008618

[102] Arifuzzaman M, Collins N, Guo C-J, Artis D (2024) Nutritional regulation of microbiota-derived metabolites: implications for immunity and inflammation. Immunity 57:14–27. 10.1016/j.immuni.2023.12.009 38198849 10.1016/j.immuni.2023.12.009 PMC10795735

[103] McCallum G, Tropini C (2024) The gut microbiota and its biogeography. Nat Rev Microbiol 22:105–118. 10.1038/s41579-023-00969-0 37740073 10.1038/s41579-023-00969-0

[104] Pi H, Sun R, McBride JR et al (2023) Clostridioides difficile ferrosome organelles combat nutritional immunity. Nature 623:1009–1016. 10.1038/s41586-023-06719-9 37968387 10.1038/s41586-023-06719-9 PMC10822667

[105] Rodionov DA, Vitreschak AG, Mironov AA, Gelfand MS (2003) Comparative genomics of the vitamin B12 metabolism and regulation in prokaryotes. J Biol Chem 278:41148–41159. 10.1074/jbc.M305837200 12869542 10.1074/jbc.M305837200

[106] Lam KN, Alexander M, Turnbaugh PJ (2019) Precision medicine goes microscopic: engineering the microbiome to improve drug outcomes. Cell Host Microbe 26:22–34. 10.1016/j.chom.2019.06.011 31295421 10.1016/j.chom.2019.06.011 PMC6709864

[107] Schirmer M, Stražar M, Avila-Pacheco J et al (2024) Linking microbial genes to plasma and stool metabolites uncovers host-microbial interactions underlying ulcerative colitis disease course. Cell Host Microbe 32:209-226.e7. 10.1016/j.chom.2023.12.013 38215740 10.1016/j.chom.2023.12.013 PMC10923022

[108] Holmes E, Li JV, Marchesi JR, Nicholson JK (2012) Gut microbiota composition and activity in relation to host metabolic phenotype and disease risk. Cell Metab 16:559–564. 10.1016/j.cmet.2012.10.007 23140640 10.1016/j.cmet.2012.10.007

[109] Visconti A, Le Roy CI, Rosa F et al (2019) Interplay between the human gut microbiome and host metabolism. Nat Commun 10:4505. 10.1038/s41467-019-12476-z 31582752 10.1038/s41467-019-12476-z PMC6776654

[110] Chen H, New P-K, Yang Y et al (2019) A forward chemical genetic screen reveals gut microbiota metabolites that modulate host physiology. Cell 177:1217-1231.e18. 10.1016/j.cell.2019.03.036 31006530 10.1016/j.cell.2019.03.036 PMC6536006

[111] Li Y, Innocentin S, Withers DR et al (2011) Exogenous stimuli maintain intraepithelial lymphocytes via aryl hydrocarbon receptor activation. Cell 147:629–640. 10.1016/j.cell.2011.09.025 21999944 10.1016/j.cell.2011.09.025

[112] Buffa JA, Romano KA, Copeland MF et al (2022) The microbial gbu gene cluster links cardiovascular disease risk associated with red meat consumption to microbiota L-carnitine catabolism. Nat Microbiol 7:73–86. 10.1038/s41564-021-01010-x 34949826 10.1038/s41564-021-01010-x PMC8732312

[113] Tang WHW, Wang Z, Levison BS et al (2013) Intestinal microbial metabolism of phosphatidylcholine and cardiovascular risk. N Engl J Med 368:1575–1584. 10.1056/NEJMoa1109400 23614584 10.1056/NEJMoa1109400 PMC3701945

[114] Wang Z, Klipfell E, Bennett BJ et al (2011) Gut flora metabolism of phosphatidylcholine promotes cardiovascular disease. Nature 472:57–63. 10.1038/nature09922 21475195 10.1038/nature09922 PMC3086762

[115] Koeth RA, Wang Z, Levinson BS et al (2013) Intestinal microbiota metabolism of L-carnitine, a nutrient in red meat, promotes atherosclerosis. Nat Med 19:576–585. 10.1038/nm.3145 23563705 10.1038/nm.3145 PMC3650111

[116] Gao F, Lv Y-W, Long J et al (2019) Butyrate improves the metabolic disorder and get microbiome dysbiosis in mice induced by a high-fat diet. Front Pharmacol 10:1040. 10.3389/fphar.2019.01040 31607907 10.3389/fphar.2019.01040 PMC6761375

[117] Perry RJ, Peng L, Barry NA et al (2016) Acetate mediates a microbiome-brain-β-cell axis to promote metabolic syndrome. Nature 534:213–217. 10.1038/nature18309 27279214 10.1038/nature18309 PMC4922538

[118] Xu Y-H, Gao C-L, Guo H-L et al (2018) Sodium butyrate supplementation ameliorates diabetic inflammation in db/db mice. J Endocrinol 238:231–244. 10.1530/JOE-18-0137 29941502 10.1530/JOE-18-0137

[119] Bouter K, Bakker GJ, Levin E et al (2018) Differential metabolic effects of oral butyrate treatment in lean versus metabolic syndrome subjects. Clin Transl Gastroenterol 9:155. 10.1038/s41424-018-0025-4 29799027 10.1038/s41424-018-0025-4 PMC5968024

[120] Canfora EE, van der Beek CM, Jocken JWE et al (2017) Colonic infusions of short-chain fatty acid mixtures promote energy metabolism in overweight/obese men: a randomized crossover trial. Sci Rep 7:2360. 10.1038/s41598-017-02546-x 28539646 10.1038/s41598-017-02546-x PMC5443817

[121] van der Beek CM, Canfora EE, Lenaerts K et al (2016) Distal, not proximal, colonic acetate infusions promote fat oxidation and improve metabolic markers in overweight/obese men. Clin Sci (Lond) 130:2073–2082. 10.1042/CS20160263 27439969 10.1042/CS20160263

[122] de Groot PF, Nikolic T, Imangaliyev S et al (2020) Oral butyrate does not affect innate immunity and islet autoimmunity in individuals with longstanding type 1 diabetes: a randomised controlled trial. Diabetologia 63:597–610. 10.1007/s00125-019-05073-8 31915895 10.1007/s00125-019-05073-8

[123] Tougaard NH, Frimodt-Møller M, Salmenkari H et al (2022) Effects of butyrate supplementation on inflammation and kidney parameters in type 1 diabetes: a randomized, double-blind, placebo-controlled trial. J Clin Med 11:3573. 10.3390/jcm11133573 35806857 10.3390/jcm11133573 PMC9267418

[124] Howard EJ, Lam TKT, Duca FA (2022) The gut microbiome: connecting diet, glucose homeostasis, and disease. Annu Rev Med 73:469–481. 10.1146/annurev-med-042220-012821 34678047 10.1146/annurev-med-042220-012821

[125] Martinez TM, Meyer RK, Duca FA (2021) Therapeutic potential of various plant-based fibers to improve energy homeostasis via the gut microbiota. Nutrients 13:3470. 10.3390/nu13103470 34684471 10.3390/nu13103470 PMC8537956

[126] Byndloss MX, Olsan EE, Rivera-Chávez F et al (2017) Microbiota-activated PPAR-γ signaling inhibits dysbiotic Enterobacteriaceae expansion. Science 357:570–575. 10.1126/science.aam9949 28798125 10.1126/science.aam9949 PMC5642957

[127] De Vadder F, Kovatcheva-Datchary P, Goncalves D et al (2014) Microbiota-generated metabolites promote metabolic benefits via gut-brain neural circuits. Cell 156:84–96. 10.1016/j.cell.2013.12.016 24412651 10.1016/j.cell.2013.12.016

[128] Frost G, Sleeth ML, Sahuri-Arisoylu M et al (2014) The short-chain fatty acid acetate reduces appetite via a central homeostasis mechanism. Nat Commun 5:3611. 10.1038/ncomms4611 24781306 10.1038/ncomms4611 PMC4015327

[129] Shimizu H, Masujima Y, Ushiroda C et al (2019) Dietary short-chain fatty acid intake improves the hepatic metabolic condition via FFAR3. Sci Rep 9:16574. 10.1038/s41598-019-53242-x 31719611 10.1038/s41598-019-53242-x PMC6851370

[130] McNelis JC, Lee YS, Mayoral R et al (2015) GPR43 potentiates β-cell function in obesity. Diabetes 64:3203–3217. 10.2337/db14-1938 26023106 10.2337/db14-1938 PMC4542437

[131] Li Z, Yi C-X, Katiraei S et al (2018) Butyrate reduces appetite and activates brown adipose tissue via the gut-brain neural circuit. Gut 67:1269–1279. 10.1136/gutjnl-2017-314050 29101261 10.1136/gutjnl-2017-314050

[132] Fogelson KA, Dorrestein PC, Zarrinpar A, Knight R (2023) The gut microbial bile acid modulation and its relevance to digestive health and diseases. Gastroenterology 164:1069–1085. 10.1053/j.gastro.2023.02.022 36841488 10.1053/j.gastro.2023.02.022 PMC10205675

[133] Wahlström A, Sayin SI, Marschall H-U, Bäckhed F (2016) Intestinal crosstalk between bile acids and microbiota and its impact on host metabolism. Cell Metab 24:41–50. 10.1016/j.cmet.2016.05.005 27320064 10.1016/j.cmet.2016.05.005

[134] Makki K, Brolin H, Petersen N et al (2023) 6α-hydroxylated bile acids mediate TGR5 signalling to improve glucose metabolism upon dietary fiber supplementation in mice. Gut 72:314–324. 10.1136/gutjnl-2021-326541 35697422 10.1136/gutjnl-2021-326541 PMC9872241

[135] Waise TMZ, Lim Y-M, Danaea Z, Zhang S-Y, Lam TKT (2021) Small intestinal taurochenodeoxycholic acid-FXR axis alters local nutrient-sensing glucoregulatory pathways in rats. Mol Metab 44:101132. 10.1016/j.molmet.2020.101132 33264656 10.1016/j.molmet.2020.101132 PMC7753965

[136] Zhang S-Y, Li RJW, Lim Y-M et al (2021) FXR in the dorsal vagal complex is sufficient and necessary for upper small intestinal microbiome-mediated changes of TCDCA to alter insulin action in rats. Gut 70:1675–1683. 10.1136/gutjnl-2020-321757 33087489 10.1136/gutjnl-2020-321757

[137] Scott SA, Fu J, Change PV (2020) Microbial tryptophan metabolites regulate gut barrier function via the aryl hydrocarbon receptor. Proc Natl Acad Sci U S A 117:19376–19387 32719140 10.1073/pnas.2000047117 PMC7431026

[138] Liu W-C, Chen P-H, Chen L-W (2020) Supplementation of endogenous AHR ligands reverses insulin resistance and associated inflammation in an insulin-dependent diabetic mouse model. J Nutr Biochem 83:108384. 10.1016/j.jnutbio.2020.108384 32512500 10.1016/j.jnutbio.2020.108384

[139] Yoo W, Zieba JK, Foegeding NJ et al (2021) High-fat diet-induced colonocyte dysfunction escalates microbiota-derived trimethylamine *N*-oxide. Science 373:813–818. 10.1126/science.aba3683 34385401 10.1126/science.aba3683 PMC8506909

[140] Koh A, Molinaro A, Ståhlman M et al (2018) Microbially produced imidazole propionate impairs insulin signaling through mTORC1. Cell 175:947-961.E17. 10.1016/j.cell.2018.09.055 30401435 10.1016/j.cell.2018.09.055

[141] Mishra SP, Wang B, Jain S et al (2023) A mechanism by which gut microbiota elevates permeability and inflammation in obese/diabetic mice and human gut. Gut 72:1848–1865. 10.1136/gutjnl-2022-327365 36948576 10.1136/gutjnl-2022-327365 PMC10512000

[142] Camillera M, Vella A (2022) What to do about leaky gut. Gut 71:424–435. 10.1136/gutjnl-2021-325428 34509978 10.1136/gutjnl-2021-325428 PMC9028931

[143] Depommier C, Everard A, Druart C et al (2019) Supplementation with Akkermansia muciniphila in overweight and obese human volunteers: a proof-of-concept exploratory study. Nat Med 25:1096–1103. 10.1038/s41591-019-0495-2 31263284 10.1038/s41591-019-0495-2 PMC6699990

[144] Dao MC, Everard A, Aron-Wisnewsky J et al (2016) Akkermansia muciniphila and improved metabolic health during a dietary intervention in obesity: relationship with gut microbiome richness and ecology. Gut 65:426–436. 10.1136/gutjnl-2014-308778 26100928 10.1136/gutjnl-2014-308778

[145] Yoon HS, Cho CH, Yun MS et al (2021) Akkermansia muciniphila secretes a glucagon-like peptide-1-inducing protein that improves glucose homeostasis and ameliorates metabolic disease in mice. Nat Microbiol 6:563–573. 10.1038/s41564-021-00880-5 33820962 10.1038/s41564-021-00880-5

[146] Plovier H, Everard A, Druart C et al (2017) A purified membrane protein from Akkermansia muciniphila or the pasteurized bacterium improves metabolism in obese and diabetic mice. Nat Med 23:107–113. 10.1038/nm.4236 27892954 10.1038/nm.4236

[147] Clasen SJ, Bell MEW, Borbón A et al (2023) Silent recognition of flagellins from human gut commensal bacteria by Toll-like receptor 5. Sci Immunol 8:eabq7001. 10.1126/sciimmunol.abq7001 36608151 10.1126/sciimmunol.abq7001

[148] Rohm TV, Fuchs R, Müller RL et al (2021) Obesity in humans is characterized by gut inflammation as shown by pro-inflammatory intestinal macrophage accumulation. Front Immunol 12:668654. 10.3389/fimmu.2021.668654 34054838 10.3389/fimmu.2021.668654 PMC8158297

[149] Monteiro-Sepulveda M, Touch S, Mendes-Sá C et al (2015) Jejunal T cell inflammation in human obesity correlated with decreased enterocyte insulin signaling. Cell Metab 22:113–124. 10.1016/j.cmet.2015.05.020 26094890 10.1016/j.cmet.2015.05.020

[150] Luck H, Khan S, Kim JH et al (2019) Gut-associated IgA^+^ immune cells regulate obesity-related insulin resistance. Nat Commun 10:3650. 10.1038/s41467-019-11370-y 31409776 10.1038/s41467-019-11370-y PMC6692361

[151] Lin Y, Xu Z, Yeoh YK et al (2023) Combing fecal microbial community data to identify consistent obesity-specific microbial signatures and shared metabolic pathways. iScience 26:106476. 10.1016/j.isci.2023.106476 37096041 10.1016/j.isci.2023.106476 PMC10122048

[152] Truong DT, Tett A, Pasolli E, Huttenhower C, Segata N (2017) Microbial strain-level population structure and genetic diversity from metagenomes. Genome Res 27:626–638. 10.1101/gr.216242.116 28167665 10.1101/gr.216242.116 PMC5378180

[153] Li J, Zhao F, Wang Y et al (2017) Gut microbiota dysbiosis contributes to the development of hypertension. Microbiome 5:14. 10.1186/s40168-016-0222-x 28143587 10.1186/s40168-016-0222-x PMC5286796

[154] Walter J, Armet AM, Finlay BB, Shanahan F (2020) Establishing or exaggerating causality for the gut microbiome: lessons from human microbiota-associated rodents. Cell 180:221–232. 10.1016/j.cell.2019.12.025 31978342 10.1016/j.cell.2019.12.025

[155] Sanz Y (2023) Turning cooperative bacteria into probiotics for human health. Nature 620:283–284. 10.1038/d41586-023-02407-w 37532854 10.1038/d41586-023-02407-w

[156] Lagkouvardos I, Pukall R, Abt B et al (2016) The Mouse Intestinal Bacterial Collection (miBC) provides host-specific insight into cultured diversity and functional potential of the gut microbiota. Nat Microbiol 1:16131. 10.1038/nmicrobiol.2016.131 27670113 10.1038/nmicrobiol.2016.131

[157] Hosomi K, Saito M, Park J et al (2022) Oral administration of Blautia wexlerae ameliorates obesity and type 2 diabetes via metabolic remodeling of the gut microbiota. Nat Commun 13:4477. 10.1038/s41467-022-32015-7 35982037 10.1038/s41467-022-32015-7 PMC9388534

[158] Romaní-Pérez M, López-Almela I, Bullich-Vilarrubias C et al (2021) Holdemanella biformis improves glucose tolerance and regulates GLP-1 signaling in obese mice. FASEB J 35:e21734. 10.1096/fj.202100126R 34143451 10.1096/fj.202100126R

[159] Chaudhari SN, McCurry MD, Devlin AS (2021) Chains of evidence from correlations to causal molecules in microbiome-linked diseases. Nat Chem Biol 17:1046–1056. 10.1038/s41589-021-00861-z 34552222 10.1038/s41589-021-00861-z PMC8480537

[160] Hajjo H, Geva-Zatorsky N (2021) Strain-level immunomodulatory variation of gut bacteria. FEBS Lett 595:1322–1327. 10.1002/1873-3468.14057 33570779 10.1002/1873-3468.14057

[161] Blandino G, Inturri R, Lazzara F, Di Rosa M, Malaguarnera L (2016) Impact of gut microbiota on diabetes mellitus. Diabetes Metab 42:303–315. 10.1016/j.diabet.2016.04.004 27179626 10.1016/j.diabet.2016.04.004

[162] Zembroski AS, D’Aquila T, Buhman KK (2021) Characterization of cytoplasmic lipid droplets in each region of the small intestine of lean and diet-induced obese mice in response to dietary fat. Am J Physiol Gastrointest Liver Physiol 321:G75–G86. 10.1152/ajpgi.00084.2021 34009042 10.1152/ajpgi.00084.2021 PMC8321799

[163] Kondo H, Minegishi Y, Komine Y et al (2006) Differential regulation of intestinal lipid metabolism-related genes in obesity-resistant A/J vs. obesity-prone C57BL/6J mice. Am J Physiol Endocrinol Metab 291:E1092–E1099. 10.1152/ajpendo.00583.2005 16822957 10.1152/ajpendo.00583.2005

[164] Martinez-Guryn K, Hubert N, Frazier K et al (2018) Small intestine microbiota regulate host digestive and absorptive adaptive responses to dietary lipids. Cell Host Microbe 23:458-469.e5. 10.1016/j.chom.2018.03.011 29649441 10.1016/j.chom.2018.03.011 PMC5912695

[165] El Aidy S, Merrifield CA, Derrien M et al (2013) The gut microbiota elicits a profound metabolic reorientation in the mouse jejunal mucosa during conventionalisation. Gut 62:1306–1314. 10.1136/gutjnl-2011-301955 22722618 10.1136/gutjnl-2011-301955

[166] Araújo JR, Tazi A, Burlen-Defranoux O et al (2020) Fermentation products of commensal bacteria alter enterocyte lipid metabolism. Cell Host Microbe 27:358-375.e7. 10.1016/j.chom.2020.01.028 32101704 10.1016/j.chom.2020.01.028

[167] Bauer PV, Duca FA, Waise TMZ et al (2018) Lactobacillus gasseri in the upper small intestine impacts an ACSL3-dependent fatty acid-sensing pathway regulating whole-body glucose homeostasis. Cell Metab 27:572-587.e6. 10.1016/j.cmet.2018.01.013 29514066 10.1016/j.cmet.2018.01.013

[168] Weninger SN, Herman C, Meyer RK et al (2023) Oligofructose improves small intestinal lipid-sensing mechanisms via alterations to the small intestinal microbiota. Microbiome 11:169. 10.1186/s40168-023-01590-2 37533066 10.1186/s40168-023-01590-2 PMC10394784

[169] Johnson AJ, Vangay P, Al-Ghalith GA et al (2019) Daily sampling reveals personalized diet-microbiome associations in humans. Cell Host Microbe 25:789-802.e5. 10.1016/j.chom.2019.05.005 31194939 10.1016/j.chom.2019.05.005

[170] Martchenko SE, Martchenko A, Cox BJ et al (2020) Circadian GLP-1 secretion in mice is dependent on the intestinal microbiome for maintenance of diurnal metabolic homeostasis. Diabetes 69:2589–2602. 10.2337/db20-0262 32928871 10.2337/db20-0262

[171] Reitmeier S, Kiessling S, Clavel T et al (2020) Arrhythmic gut microbiome signatures predict risk of type 2 diabetes. Cell Host Microbe 28:258-272.e6. 10.1016/j.chom.2020.06.004 32619440 10.1016/j.chom.2020.06.004

[172] Brettle H, Tran V, Drummond GR et al (2022) Sex hormones, intestinal inflammation, and the gut microbiome: major influencers of the sexual dimorphisms in obesity. Front Immunol 13:971048. 10.3389/fimmu.2022.971048 36248832 10.3389/fimmu.2022.971048 PMC9554749

[173] Markle JGM, Frank DN, Mortin-Toth S et al (2013) Sex differences in the gut microbiome drive hormone-dependent regulation of autoimmunity. Science 339:1084–1088. 10.1126/science.1233521 23328391 10.1126/science.1233521

[174] Wang D, Liu J, Zhou L, Zhang Q, Li M, Xiao X (2022) Effects of oral glucose-lowering agents on gut microbiota and microbial metabolites. Front Endocrinol (Lausanne) 13:905171. 10.3389/fendo.2022.905171 35909556 10.3389/fendo.2022.905171 PMC9326154

[175] Bäckhed F, Manchester JK, Semenkovich CF, Gordon JI (2007) Mechanisms underlying the resistance to diet-induced obesity in germ-free mice. Proc Natl Acad Sci U S A 104:979–984. 10.1073/pnas.0605374104 17210919 10.1073/pnas.0605374104 PMC1764762

[176] Cheng AG, Ho P-Y, Aranda-Díaz A et al (2022) Design, construction, and in vivo augmentation of a complex gut microbiome. Cell 185:3617-3636.e19. 10.1016/j.cell.2022.08.003 36070752 10.1016/j.cell.2022.08.003 PMC9691261

[177] Zhang C, Yin A, Li H et al (2015) Dietary modulation of gut microbiota contributes to alleviation of both genetic and simple obesity in children. EBioMedicine 2:968–984. 10.1016/j.ebiom.2015.07.007 26425705 10.1016/j.ebiom.2015.07.007 PMC4563136

[178] Fei N, Zhao L (2013) An opportunistic pathogen isolated from the gut of an obese human causes obesity in germfree mice. ISME J 7:880–884. 10.1038/ismej.2012.153 23235292 10.1038/ismej.2012.153 PMC3603399

[179] Fei N, Bruneau A, Zhang X et al (2020) Endotoxin producers overgrowing in human gut microbiota as the causative agents for nonalcoholic fatty liver disease. mBio 11:e03263-19. 10.1128/mBio.03263-19 32019793 10.1128/mBio.03263-19 PMC7002352

[180] Cani PD, Amar J, Iglesias MA et al (2007) Metabolic endotoxemia initiates obesity and insulin resistance. Diabetes 56:1761–1772. 10.2337/db06-1491 17456850 10.2337/db06-1491

[181] Logan IE, Bobe G, Miranda CL et al (2020) Germ-free Swiss Webster mice on a high-fat diet develop obesity, hyperglycemia, and dyslipidemia. Microorganisms 8:520. 10.3390/microorganisms8040520 32260528 10.3390/microorganisms8040520 PMC7232377

[182] Almind K, Kahn CR (2004) Genetic determinants of energy expenditure and insulin resistance in diet-induced obesity in mice. Diabetes 53:3274–3285. 10.2337/diabetes.53.12.3274 15561960 10.2337/diabetes.53.12.3274

[183] Pang X, Hua X, Yang Q et al (2007) Inter-species transplantation of gut microbiota from human to pigs. ISME J 1:156–162. 10.1038/ismej.2007.23 18043625 10.1038/ismej.2007.23

[184] Rawls JF, Samuel BS, Gordon JI (2004) Gnotobiotic zebrafish reveal evolutionarily conserved responses to the gut microbiota. Proc Natl Acad Sci U S A 101:4596–4601. 10.1073/pnas.0400706101 15070763 10.1073/pnas.0400706101 PMC384792

[185] Melancon E, Gomez De La Torre Canny S, Sichel S et al (2017) Best practices for germ-free derivation and gnotobiotic zebrafish husbandry. Methods Cell Biol 138:61–100. 10.1016/bs.mcb.2016.11.005 28129860 10.1016/bs.mcb.2016.11.005 PMC5568843

[186] Leung CM, de Haan P, Ronaldson-Bouchard K et al (2022) A guide to the organ-on-a-chip. Nat Rev Methods Primers 2:33. 10.1038/s43586-022-00118-6

[187] Jalili-Firoozinezhad S, Gazzaniga FS, Calamari EL et al (2019) A complex human gut microbiome cultured in an anaerobic intestine-on-a-chip. Nat Biomed Eng 3:520–531. 10.1038/s41551-019-0397-0 31086325 10.1038/s41551-019-0397-0 PMC6658209

[188] Trapecar M, Wogram E, Svoboda D et al (2021) Human physiomimetic model integrating microphysiological systems of the gut, liver, and brain for studies of neurodegenerative diseases. Sci Adv 7:eabd1707. 10.1126/sciadv.abd1707 33514545 10.1126/sciadv.abd1707 PMC7846169

[189] Yang D, Park SY, Park YS, Eun H, Lee SY (2020) Metabolic engineering of Escherichia coli for natural product biosynthesis. Trends Biotechnol 38:745–765. 10.1016/j.tibtech.2019.11.007 31924345 10.1016/j.tibtech.2019.11.007

[190] Fang H, Li D, Kang J, Jiang P, Sun J, Zhang D (2018) Metabolic engineering of Escherichia coli for de novo biosynthesis of vitamin B_12_. Nat Commun 9:4917. 10.1038/s41467-018-07412-6 30464241 10.1038/s41467-018-07412-6 PMC6249242

[191] Kolisnychenko V, Plunkett G 3rd, Herring CD et al (2002) Engineering a reduced Escherichia coli genome. Genome Res 12:640–647. 10.1101/gr.217202 11932248 10.1101/gr.217202 PMC187512

[192] Tan Y, Liang J, Lai M, Wan S, Luo X, Li F (2023) Advances in synthetic biology toolboxes paving the way for mechanistic understanding and strain engineering of gut commensal Bacteroides spp. and Clostridium spp. Biotechnol Adv 69:108272. 10.1016/j.biotechadv.2023.108272 37844770 10.1016/j.biotechadv.2023.108272

[193] Whitaker WR, Shepherd ES, Sonnenburg JL (2017) Tunable expression tools enable single-cell strain distinction in the gut microbiome. Cell 169:538-546.e12. 10.1016/j.cell.2017.03.041 28431251 10.1016/j.cell.2017.03.041 PMC5576361

[194] Jumper J, Evans R, Pritzel A et al (2021) Highly accurate protein structure prediction with AlphaFold. Nature 596:583–589. 10.1038/s41586-021-03819-2 34265844 10.1038/s41586-021-03819-2 PMC8371605

[195] Varadi M, Anyango S, Deshpande M et al (2022) AlphaFold Protein Structure Database: massively expanded the structural coverage of protein-sequence space with high-accuracy models. Nucleic Acids Res 50:D439–D444. 10.1093/nar/gkab1061 34791371 10.1093/nar/gkab1061 PMC8728224

[196] Wu G, Zhao N, Zhang C, Lam YY, Zhao L (2021) Guild-based analysis for understanding gut microbiome in human health and diseases. Genome Med 13:22. 10.1186/s13073-021-00840-y 33563315 10.1186/s13073-021-00840-y PMC7874449

[197] Bowers RM, Kyrpides NC, Stepanauskas R et al (2017) Minimum information about a single amplified genome (MISAG) and a metagenome-assembled genome (MIMAG) of bacteria and archaea. Nat Biotechnol 35:725–731. 10.1038/nbt.3893 28787424 10.1038/nbt.3893 PMC6436528

[198] Shalon D, Culver RN, Grembi JA et al (2023) Profiling the human intestinal environment under physiological conditions. Nature 617:581–591. 10.1038/s41586-023-05989-7 37165188 10.1038/s41586-023-05989-7 PMC10191855

[199] Donia MS, Fischbach MA (2015) Small molecules from the human microbiota. Science 349:1254766. 10.1126/science.1254766 26206939 10.1126/science.1254766 PMC4641445

[200] Zhai L, Xiao H, Lin C et al (2023) Gut microbiota-derived tryptamine and phenethylamine impair insulin sensitivity in metabolic syndrome and irritable bowel syndrome. Nat Commun 14:4986. 10.1038/s41467-023-40552-y 37591886 10.1038/s41467-023-40552-y PMC10435514

[201] Vatanen T, Franzosa EA, Schwager R et al (2018) The human gut microbiome in early-onset type 1 diabetes from the TEDDY study. Nature 562:589–594. 10.1038/s41586-018-0620-2 30356183 10.1038/s41586-018-0620-2 PMC6296767

[202] Berry D, Loy A (2018) Stable-isotope probing of human and animal microbiome function. Trends Microbiol 26:999–1007. 10.1016/j.tim.2018.06.004 30001854 10.1016/j.tim.2018.06.004 PMC6249988

[203] Reynolds AN, Akerman AP, Mann J (2020) Dietary fibre and whole grains in diabetes management: systematic review and meta-analysis. PLoS Med 17:e1003053. 10.1371/journal.pmed.1003053 32142510 10.1371/journal.pmed.1003053 PMC7059907

[204] Chandalia M, Garg A, Lutjohann D, von Bergmann K, Grundy SM, Brinkley LJ (2000) Beneficial effects of high dietary fiber intake in patients with type 2 diabetes mellitus. N Engl J Med 342:1392–1398. 10.1056/NEJM200005113421903 10805824 10.1056/NEJM200005113421903

[205] Blaak EE, Canfora EE, Theis S et al (2020) Short chain fatty acids in human gut and metabolic health. Benef Microbes 11:411–455. 10.3920/BM2020.0057 32865024 10.3920/BM2020.0057

[206] Khosravi Z, Hadi A, Tutunchi H et al (2022) The effects of butyrate supplementation on glycemic control, lipid profile, blood pressure, nitric oxide level and glutathione peroxidase activity in type 2 diabetic patients: a randomized triple-blind, placebo-controlled trial. Clin Nutr ESPEN 49:79–85. 10.1016/j.clnesp.2022.03.008 35623879 10.1016/j.clnesp.2022.03.008

[207] O’Toole PW, Marchesi JR, Hill C (2017) Next-generation probiotics: the spectrum from probiotics to live biotherapeutics. Nat Microbiol 2:17057. 10.1038/nmicrobiol.2017.57 28440276 10.1038/nmicrobiol.2017.57

[208] Wang C-H, Yen H-R, Lu W-L et al (2022) Adjuvant probiotics of *Lactobacillus salivarius* subsp. *salicinius* AP-32, *L. johnsonii* MH-68, and *Bifidobacterium animalis* subsp. *lactis* CP-9 attenuate glycemic levels and inflammatory cytokine in patients with type 1 diabetes mellitus. Front Endocrinol (Lausanne) 13:754401. 10.3389/fendo.2022.754401 35299968 10.3389/fendo.2022.754401 PMC8921459

[209] Cabrera SM, Coren AT, Pant T et al (2022) Probiotic normalization of systemic inflammation in siblings of type 1 diabetes patients: an open-label pilot study. Sci Rep 12:3306. 10.1038/s41598-022-07203-6 35228584 10.1038/s41598-022-07203-6 PMC8885673

[210] Tao Y-W, Gu Y-L, Mao X-Q, Zhang L, Pei Y-F (2020) Effects of probiotics on type II diabetes mellitus: a meta-analysis. J Transl Med 18:30. 10.1186/s12967-020-02213-2 31952517 10.1186/s12967-020-02213-2 PMC6966830

[211] Zhou Q, Zhang Y, Wang X et al (2020) Gut bacteria *Akkermansia* is associated with reduced risk of obesity: evidence from the American Gut Project. Nutr Metab (Lond) 17:90. 10.1186/s12986-020-00516-1 33110437 10.1186/s12986-020-00516-1 PMC7583218

[212] Gilijamse PW, Hartstra AV, Levin E et al (2020) Treatment with Anaerobutyricum soehngenii: a pilot study of safety and dose-response effects on glucose metabolism in human subjects with metabolic syndrome. NPJ Biofilms Microbiomes 6:16. 10.1038/s41522-020-0127-0 32221294 10.1038/s41522-020-0127-0 PMC7101376

[213] Koopen A, Witjes J, Wortelboer K et al (2022) Duodenal *Anaerobutyricum soehngenii* infusion stimulates GLP-1 production, ameliorates glycaemic control and beneficially shapes the duodenal transcriptome in metabolic syndrome subjects: a randomized double-blind placebo-controlled cross-over study. Gut 71:1577–1587. 10.1136/gutjnl-2020-323297 34697034 10.1136/gutjnl-2020-323297 PMC9279853

[214] Hanssen NMJ, do Vos WM, Nieuwdorp M (2021) Fecal microbiota transplantation in human metabolic diseases: from a murky past to a bright future? Cell Metab 33:1098–1110. 10.1016/j.cmet.2021.05.005 34077717 10.1016/j.cmet.2021.05.005

[215] Pellegrini S, Sordi V, Bolla AM et al (2017) Duodenal mucosa of patients with type 1 diabetes shows distinctive inflammatory profile and microbiota. J Clin Endocrinol Metab 102:1468–1477. 10.1210/jc.2016-3222 28324102 10.1210/jc.2016-3222

[216] Vrieze A, Van Nood E, Holleman F et al (2012) Transfer of intestinal microbiota from lean donors increases insulin sensitivity in individuals with metabolic syndrome. Gastroenterology 143:913-916.e7. 10.1053/j.gastro.2012.06.031 22728514 10.1053/j.gastro.2012.06.031

[217] Kootte RS, Levin E, Salojärvi J et al (2017) Improvement of insulin sensitivity after lean donor feces in metabolic syndrome is driven by baseline intestinal microbiota composition. Cell Metab 26:611-619.e6. 10.1016/j.cmet.2017.09.008 28978426 10.1016/j.cmet.2017.09.008

[218] Schmidt TSB, Li SS, Maistrenko OM et al (2022) Drivers and determinants of strain dynamics following fecal microbiota transplantation. Nat Med 28:1902–1912. 10.1038/s41591-022-01913-0 36109636 10.1038/s41591-022-01913-0 PMC9499871

[219] de Groot P, Nikolic T, Pellegrinin S et al (2021) Faecal microbiota transplantation halts progression of human new-onset type 1 diabetes in a randomised controlled trial. Gut 70:92–105. 10.1136/gutjnl-2020-322630 33106354 10.1136/gutjnl-2020-322630 PMC7788262

[220] Rinott E, Youngster I, Meir AY et al (2021) Effects of diet-modulated autologous fecal microbiota transplantation on weight regain. Gastroenterology 160:158-173.e10. 10.1053/j.gastro.2020.08.041 32860791 10.1053/j.gastro.2020.08.041 PMC7755729

[221] Mocanu V, Zhang Z, Deehan EC et al (2021) Fecal microbial transplantation and fiber supplementation in patients with serve obesity and metabolic syndrome: a randomized double-blind, placebo-controlled phase 2 trial. Nat Med 27:1272–1279. 10.1038/s41591-021-01399-2 34226737 10.1038/s41591-021-01399-2

[222] Witjes JJ, Smits LP, Pekmez CT et al (2020) Donor fecal microbiota transplantation alters gut microbiota and metabolites in obese individuals with steatohepatitis. Hepatol Commun 4:1578–1590. 10.1002/hep4.1601 33163830 10.1002/hep4.1601 PMC7603524

[223] Ng SC, Xu Z, Mak JWY et al (2022) Microbiota engraftment after faecal microbiota transplantation in obese subjects with type 2 diabetes: a 24-week, double-blind, randomized controlled trial. Gut 71:716–723. 10.1136/gutjnl-2020-323617 33785557 10.1136/gutjnl-2020-323617

[224] Yu EW, Gao L, Stastka P et al (2020) Fecal microbiota transplantation for the improvement of metabolism in obesity: the FMT-TRIM double-blind placebo-controlled pilot trial. PLoS Med 17:e1003051. 10.1371/journal.pmed.1003051 32150549 10.1371/journal.pmed.1003051 PMC7062239

[225] Kamer O, Rinott E, Tsaban G et al (2023) Successful weight regain attenuation by autologous fecal microbiota transplantation is associated with non-core gut microbiota changes during weight loss; randomized controlled trial. Gut Microbes 15:2264457. 10.1080/19490976.2023.2264457 37796016 10.1080/19490976.2023.2264457 PMC10557561

[226] Fessler J, Matson V, Gajewski TF (2019) Exploring the emerging role of the microbiome in cancer immunotherapy. J Immunother Cancer 7:108. 10.1186/s40425-019-0574-4 30995949 10.1186/s40425-019-0574-4 PMC6471869

[227] Ianiro G, Punčochář M, Karcher N et al (2022) Variability of strain engraftment and predictability of microbiome composition after fecal microbiota transplantation across different diseases. Nat Med 28:1913–1923. 10.1038/s41591-022-01964-3 36109637 10.1038/s41591-022-01964-3 PMC9499858

[228] Attaye I, Warmbrunn MV, Boot ANAF et al (2022) A systematic review and meta-analysis of dietary interventions modulating gut microbiota and cardiometabolic diseases: striving for new standards in microbiome studies. Gastroenterology 162:1911–1932. 10.1053/j.gastro.2022.02.011 35151697 10.1053/j.gastro.2022.02.011

[229] European Medicines Agency. Faecal microbiota transplantation: EU-IN horizon scanning report. Available from https://www.ema.europa.eu/en/documents/report/faecal-microbiota-transplantation-eu-horizon-scanning-report_en.pdf. Accessed 7 February 2024

[230] Bénard MV, de Bruijn CMA, Fenneman AC et al (2022) Challenges and costs of donor screening for fecal microbiota transplantations. PLoS One 17:e0276323. 10.1371/journal.pone.0276323 36264933 10.1371/journal.pone.0276323 PMC9584411

[231] Groen AK, Nieuwdorp M (2017) An evaluation of the therapeutic potential of fecal microbiota transplantation to treat infectious and metabolic diseases. EMBO Mol Med 9:1–3. 10.15252/emmm.201607035 27861129 10.15252/emmm.201607035 PMC5210083

[232] Pascual J, Tanner K, Vilanova C, Porcar M, Delgado A (2021) The microbial terroir: open questions on the Nagoya protocol applied to microbial resources. Microb Biotechnol 14:1878–1880. 10.1111/1751-7915.13839 34311495 10.1111/1751-7915.13839 PMC8449654

[233] Bojanova DP, Bordenstein SR (2016) Fecal transplants: what is being transferred? PLoS Biol 14:e1002503. 10.1371/journal.pbio.1002503 27404502 10.1371/journal.pbio.1002503 PMC4942072

[234] Kovatcheva-Datchary P, Tremaroli V, Bäckhed F (2013) The gut microbiota. In: Rosenberg E, DeLong EF, Lory S, Stackebrandt E, Thompson F (eds) The prokaryotes: human microbiology, 4th edn. Springer-Verlag, Berlin, Germany, pp 3–24. 10.1007/978-3-642-30144-5_87

[235] Caesar R (2019) Pharmacologic and nonpharmacologic therapies for the gut microbiota in type 2 diabetes. Can J Diabetes 43:224–231. 10.1016/j.jcjd.2019.01.007 30929665 10.1016/j.jcjd.2019.01.007

[236] Zhou Z-Y, Ren L-W, Zhan P, Yang H-Y, Chai D-D, Yu Z-W (2016) Metformin exerts glucose-lowering action in high-fat fed mice via attenuating endotoxemia and enhancing insulin signaling. Acta Pharmacol Sin 37:1063–1075. 10.1038/aps.2016.21 27180982 10.1038/aps.2016.21 PMC4973377

[237] Shin N-R, Lee J-C, Lee H-Y et al (2014) An increase in the Akkermansia spp. Population induced by metformin treatment improves glucose homeostasis in diet-induced obese mice. Gut 63:727–735. 10.1136/gutjnl-2012-303839 23804561 10.1136/gutjnl-2012-303839

