# Supplementary material for: The gut microbiota and diabetes: research, translation, and clinical applications – 2023 Diabetes, Diabetes Care, and Diabetologia Expert Forum
Source: Diabetologia. 2024 Jun 24;67(9):1760–82. doi: 10.1007/s00125-024-06198-1 (PMC11410996; doi:10.1007/s00125-024-06198-1)
Supplement: Supplementary file 1 — Supplementary file1 (PPTX 746 KB) [file 125_2024_6198_MOESM1_ESM.pptx]

## Slide 1
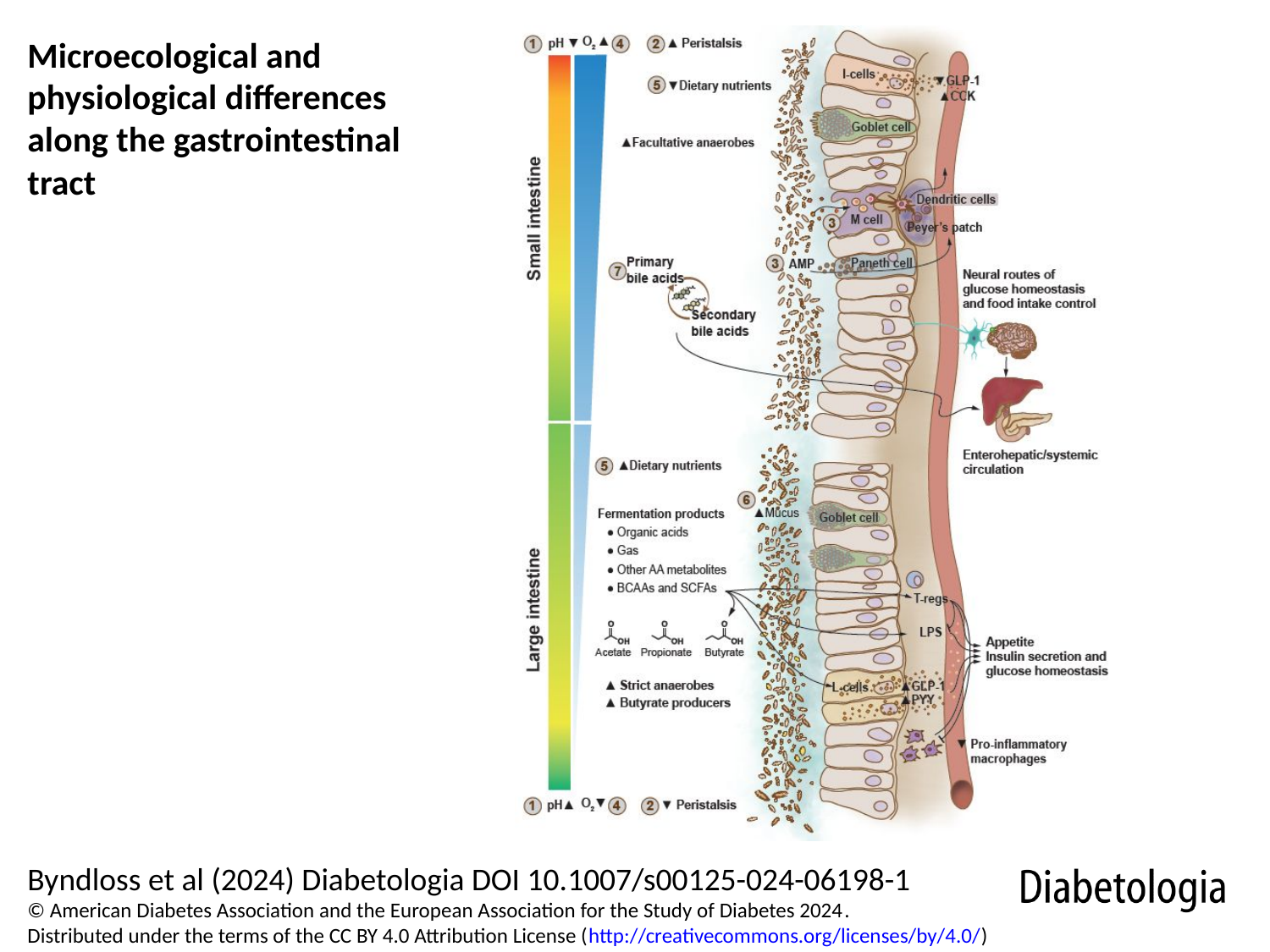

Microecological and physiological differences along the gastrointestinal tract
Byndloss et al (2024) Diabetologia DOI 10.1007/s00125-024-06198-1
© American Diabetes Association and the European Association for the Study of Diabetes 2024.
Distributed under the terms of the CC BY 4.0 Attribution License (http://creativecommons.org/licenses/by/4.0/)

## Slide 2
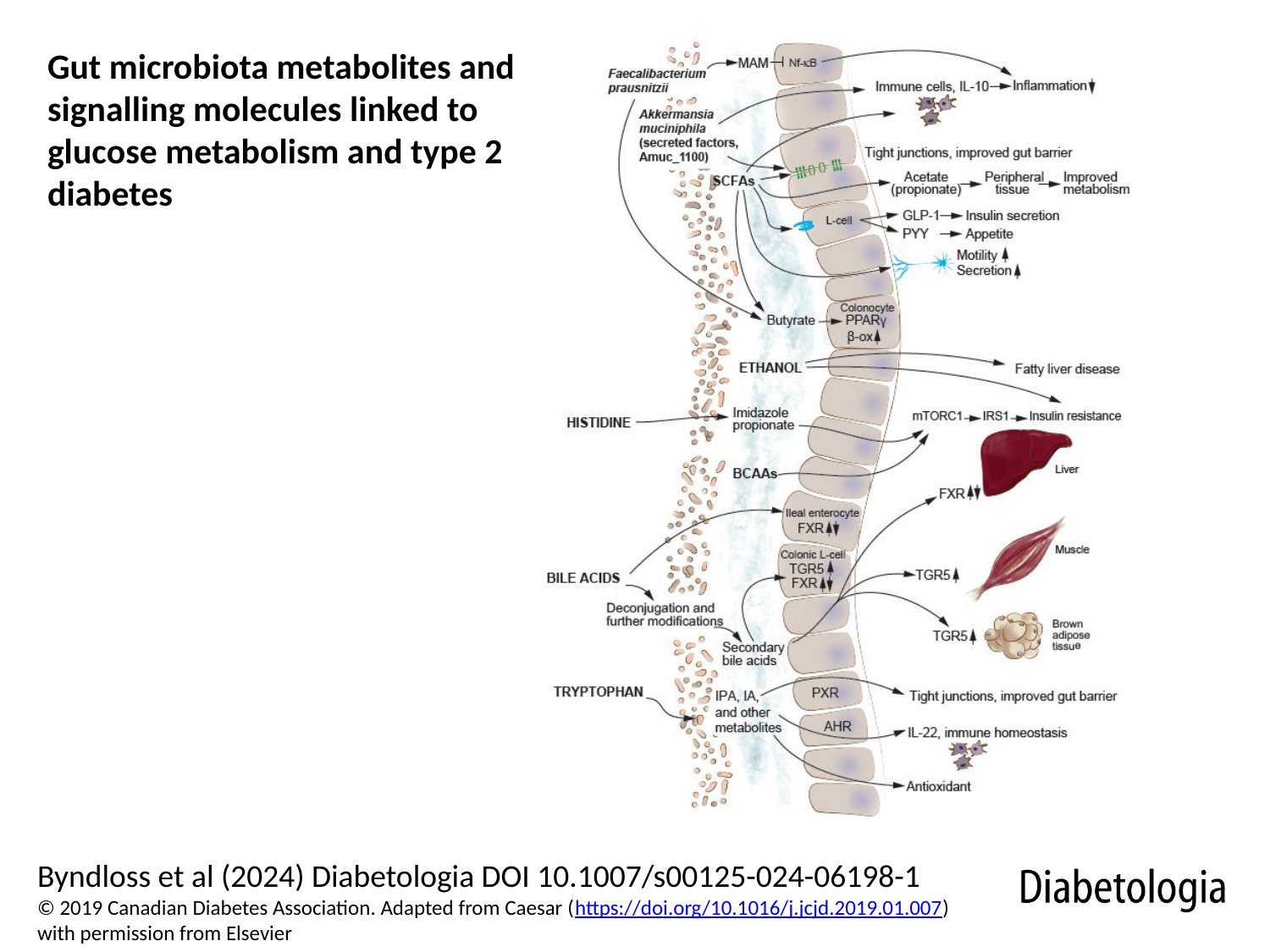

Gut microbiota metabolites and signalling molecules linked to glucose metabolism and type 2 diabetes
Byndloss et al (2024) Diabetologia DOI 10.1007/s00125-024-06198-1
© 2019 Canadian Diabetes Association. Adapted from Caesar (https://doi.org/10.1016/j.jcjd.2019.01.007)
with permission from Elsevier

## Slide 3
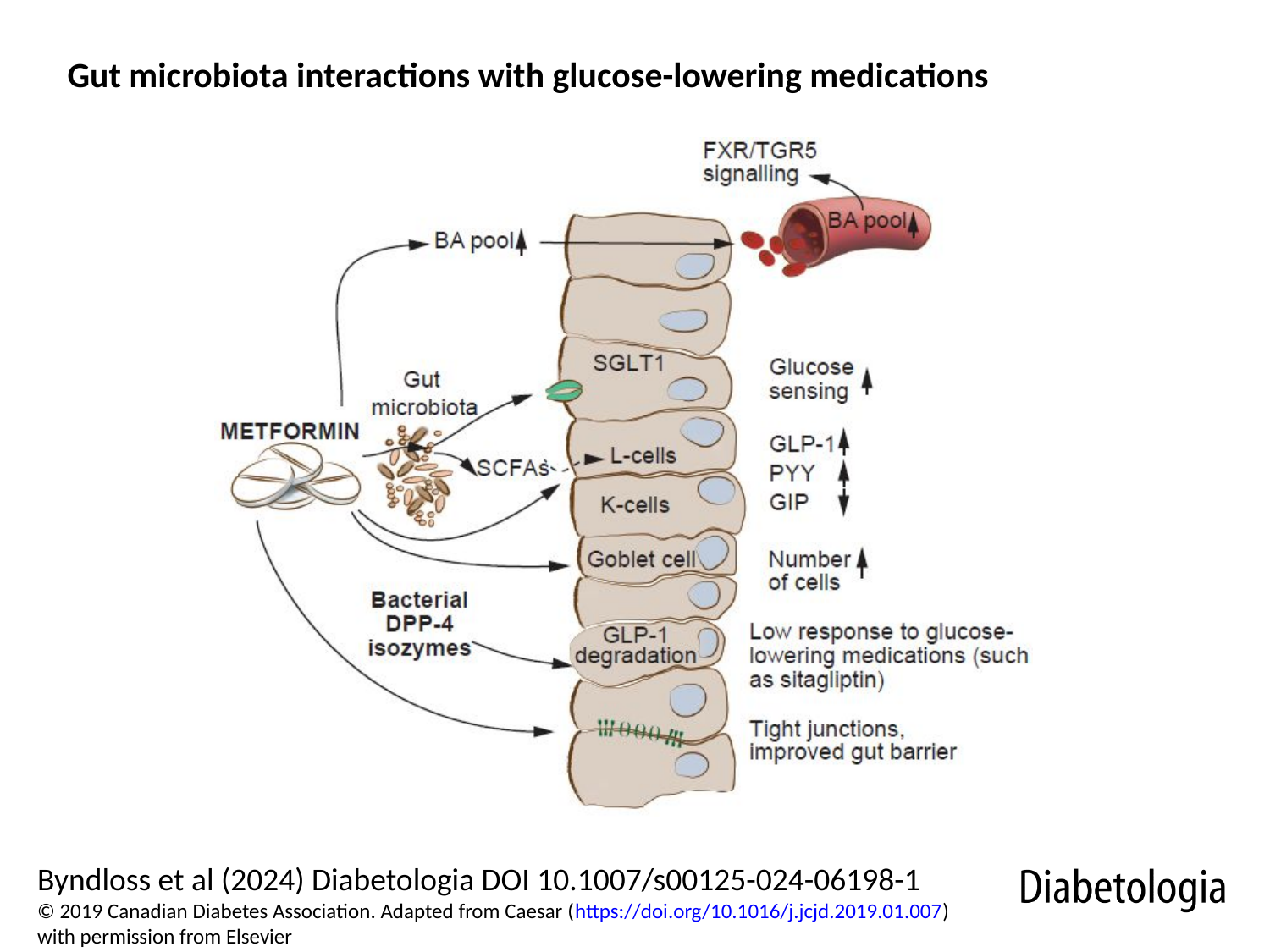

Gut microbiota interactions with glucose-lowering medications
Byndloss et al (2024) Diabetologia DOI 10.1007/s00125-024-06198-1
© 2019 Canadian Diabetes Association. Adapted from Caesar (https://doi.org/10.1016/j.jcjd.2019.01.007)
with permission from Elsevier
